# Discovery of Consistent QTLs of Wheat Spike-Related Traits under Nitrogen Treatment at Different Development Stages

**DOI:** 10.3389/fpls.2017.02120

**Published:** 2017-12-15

**Authors:** Zhiying Deng, Yong Cui, Qingdian Han, Wenqi Fang, Jifa Li, Jichun Tian

**Affiliations:** ^1^Group of Wheat Quality Breeding, State Key Laboratory of Crop Biology, Key Laboratory of Crop Biology of Shandong Province, Cooperation Innovation Centre of Efficient Production with High Annual Yield of Wheat and Corn, Agronomy College, Shandong Agricultural University, Tai'an, China; ^2^Horticulture, College of Agriculture and Forestry Science, Linyi University, Linyi, China

**Keywords:** wheat, spike-related traits, QTL mapping, molecular marker, nitrogen treatments

## Abstract

Spike-related traits such as spike length (Sl), fertile spikelet number (Fsn), sterile spikelet number (Ssn), grain number per spike (Gns), and thousand-kernel weight (Tkw) are important factors influencing wheat yield. However, reliably stable markers that can be used for molecular breeding in different environments have not yet been identified. In this study, a double haploid (DH) population was used for quantitative trait locus (QTL) mapping of five spike-related traits under four different nitrogen (N) supply dates in two locations and years. Seventy additive QTLs with phenotypic variation ranging from 4.12 to 34.74% and 10 major epistatic QTLs were identified. Eight important chromosomal regions on five chromosomes (1B, 2B, 2D, 5D, and 6A) were found. Sixteen stable QTLs were detected for which N application had little effect. Among those stable QTLs, *QSl.sdau-2D-1*, and *QSl.sdau-2D-2*, with phenotypic variation explained (PVE) of 10.4 and 24.2%, respectively, were flanked by markers *Xwmc112* and *Xcfd53* in the same order. The QTLs *QSsn.sdau-2B-1, QFsn.sdau-2B-1*, and *QGns.sdau-2B*, with PVE ranging from 4.37 to 28.43%, collocated in the *Xwmc179*-*Xbarc373* marker interval. The consistent kernel wheat QTL (*QTkw.sdau-6A*) on the long arm of chromosome 6A, flanked by SSR markers *Xbarc1055* and *Xwmc553*, showed PVE of 5.87–15.18%. Among these stable QTLs, the two flanking markers *Xwmc112* and *Xcfd53* have been validated using different varieties and populations for selecting Sl. Therefore, these results will be of great value for marker-assisted selection (MAS) in breeding programs and will accelerate the understanding of the genetic relationships among spike-related traits at the molecular level.

## Introduction

Wheat is one of the most important crops in the world and is critical in supporting the global population (Gupta et al., 2010). In conventional breeding, breeders typically increase wheat yield by altering the spike number per hectare, grain number per ear, or thousand-grain weight (Ma et al., 2007; Cui et al., 2012). However, due to certain shortcomings, such as a long crop cycle, high cost and poor results, significantly improving wheat yield via traditional breeding of these traits is difficult. Additionally, wheat yield and related traits are not easily studied because of the influences of many factors, including genotypic and environmental factors and their interactions (Cui et al., 2012). Important traits related to yield are spike length (Sl), fertile spikelet number (Fsn), sterile spikelet number (Ssn), grain number per spike (Gns), and thousand-kernel weight (Tkw) (Liu et al., 2016). Sl is typically regarded as a factor that indirectly affects yield through the total number of spikelets, Fsn and Gns (Ijaz and Kashif, 2013). In contrast, the other four traits are direct factors affecting wheat yield; as important breeding targets, they are consistently a focus of research. With the development of molecular markers, much research has been performed on constructing genetic maps and quantitative trait locus (QTL) mapping of wheat traits related to yield (Börner et al., 2002; Huang et al., 2006; Marza et al., 2006; Kumar et al., 2007; Li et al., 2007; Ma et al., 2007; Chu et al., 2008; Sun et al., 2009; Wang et al., 2009, 2011; Zhang et al., 2009b; Tsilo et al., 2010; Ding et al., 2011; Lu et al., 2011; Cui et al., 2012; Yang et al., 2012; Wu et al., 2014; Liu et al., 2016) that have been found to be controlled by polygenes and are thus quantitative in nature.

In recent years, extensive study of the genetics of spike-related traits has determined that QTLs identified in different materials involve many chromosomes (Yuan et al., 1997; Börner et al., 2002; Deng et al., 2005; Marza et al., 2006; Kumar et al., 2007; Li et al., 2007; Ma et al., 2007; Chu et al., 2008; Lu et al., 2011; Wang et al., 2011; Cui et al., 2012; Liu et al., 2016) and almost the entire genome in wheat.

The genetics of Sl have been studied by using traditional methods and different materials, such as durum wheat, barley, and wheat (Zheng et al., 1992; Yuan et al., 1997; Sharma et al., 2003; Gorjanović and Kraljevic-Balalic, 2005; Gorjanovic and Kraljevic-Balalic, 2007; Madiš et al., 2010; Lu et al., 2011; Nataša et al., 2014). Most of these studies show that additive genes play important roles in Sl in wheat. For instance, Deng et al. (2005) found that 10 chromosomes (4A, 5A, 6A, 7A, 1B, 3B, 4B, 5B, 6B, and 7D) significantly affect Sl. Certain genes involving five chromosomes (3A, 5A, 2B, 1D, and 6D), particularly 2B, also influence Sl (Kumar et al., 2007). Based on QTL mapping, chromosomes 1A, 1B, 4A, and 7A (Jantasuriyarat et al., 2004; Ma et al., 2007) were found to be involved in Sl in common wheat. Using conditional and unconditional QTL mapping, Yu et al. (2014) dissected the uppermost internode and Sl in two wheat recombinant inbred line (RIL) populations and found 13 QTLs affecting Sl on chromosomes 1A, 1B, 3D, 4A, 5A, 5D, 6A, 7A, 7B, and 7D, accounting for 3.3–25% of the phenotypic variation. In addition, Wu et al. (2014) conducted fine mapping of a QTL interval for Sl and grain weight in bread wheat and identified an important locus, HL1 (head length), which was mapped to a 0.9-cM interval flanked by *Xcfd53* and *DG371*.

Some major QTLs for important yield-related traits other than SLl have been reported. For example, QTLs have been identified on chromosome 5B that affect spikelet number per spike and Gns (Miura et al., 1992), and Börner et al. (2002) detected major QTLs on chromosomes 2DS, 4AL, and 5AL for Gns and grain weight per spike by studying a BC_2_F_1_ population. In addition, eight QTLs on chromosomes 1D, 2A, 3D, 6A, 7A, and 7D for Gns have been found (Huang et al., 2004), and Li et al. (2007) identified five QTLs for Ssn on chromosomes 1A, 4A, 6B, 7A, and 7D and five QTLs for Fsn on chromosomes 2A, 5D, 6B, and 7D using an RIL population. In another study, Cui et al. (2012) used two RIL populations with one common parent (Weimai 8) to map spike-related traits and simultaneously detected QTLs for Ssn on chromosomes 3D, 4A, and 7B. Importantly, they detected a pleiotropic effect of these QTLs. The interval between markers *Xcfd46* and *Xwmc702* on chromosome 7D was detected for the QTLs affecting Gns, Fsn, Sl, and total spikelet number per spike using RIL and immortalized F_2_ (IF_2_) populations (Ma et al., 2007), and Wang et al. (2011) identified QTLs for these four traits on the same marker intervals of chromosomes 4BL, 5A, and 6A using an F_2−3_ population. Deng et al. (2011) found QTLs for Gns, Sl, and total spikelet number per spike in the same marker interval of chromosome 4B. Stable major QTLs for six spike-related traits (Tkw, Sl, Gns, Fsn, Ssn, and total spikelet number per spike) were simultaneously identified in the marker interval *EX_C101685-RAC875_C27563* of chromosome 4B (Liu et al., 2016). These latter findings indicate that chromosome 4B has some important QTLs/genes for controlling spike-related traits.

Although many QTLs for these five spike-related traits in wheat have been identified, few stable molecular markers have been found that can be used for marker-assisted selection (MAS) in wheat breeding. Therefore, the objective of this study was to identify stable molecular markers for these traits that could be used in MAS by performing QTL mapping of wheat lines subjected to nitrogen (N) treatment at different development stages.

## Materials and methods

### Plant materials

A population of 168 double haploid (DH) wheat lines derived from a cross of elite cultivars Huapei 3 and Yumai 57 was used for QTL mapping. Huapei3 is an elite variety with desirable agronomic characteristics for early maturity, high yield and high resistance to several diseases, and this variety was released by Henan province in 2006. Additionally, Yumai57 is a widely-cultivated variety grown under different ecological conditions because of its yield stability, and this variety was registered by the State of China in 2003. The parents differ in several important agronomical traits as well as in baking quality parameters, as described by Zhang et al. (2008).

### Field trials

A total of 168 lines and their parents were grown in two distinct locations, Tai'an (TA), Shandong Province (36°12′N, 117°04′E), and Jiyuan (JY), Henan Province (35°05′N, 112°36′E), China, in the 2010–2011 growing season. In the 2011–2012 growing season only in TA, pot experiments were conducted using the same soil as that at the TA location.

The soil at both locations is mainly brown, and the soil properties were tested at a depth of 0–20 cm before sowing. At the TA location, the soil had an organic matter content of 17.58 g/kg (Walkley and Black, 1934), an alkali-hydrolyzable N content of 23.46 mg/kg (semi-micro Kjeldahl method; Kjeltec 8200 Auto Distillation Unit, Foss, Hillerød, Denmark; Yuen and Pollard, 1953; Bremner, 1960), a rapidly available phosphorus (P) content of 45.08 mg/kg (Olsen method; Zandstra, 1968), and an available potassium (K) content of 153.5 mg/kg (Dirks-Sheffer method; Melich, 1953). At the JY location, the contents of organic matter, alkali-hydrolyzable N, rapidly available P and K were 13.7 g/kg, 67.97 mg/kg, 29.7 mg/kg, and 137.7 mg/kg, respectively.

Four treatments were applied at JY and TA in both growing seasons. T0 was the no N fertilization treatment; T1, T2, and T3 were treatments of top-dressing with 120 kg/ha of pure N at the turning-green (GS 28–29), jointing (GS 31–32) and booting (GS 47) stages, respectively (Zadoks growth stages, Zadoks et al., 1974). The base fertilization before applying the T1, T2, and T3 treatments was 120 kg/ha of pure N. Urea (NH_2_)_2_CO was used as N fertilizer in all treatments. Plants in all treatments were watered only one time at each stage, i.e., at the turning-green (GS 28–29), jointing (GS 31–32) and booting (GS 47) stages. N fertilizer was applied during watering.

In order to obtain the accurate result, the DH lines with the same or similar developmental stages were assigned to the same group and then grown in one plot. Ultimately, the DH population was divided into eight groups according to the developmental stages, which based on the agronomic characteristics carefully investigated in our previous study (Zhang et al., 2008, 2009a,b). Nitrogen fertilization was applied in the different plots according to the developmental stages.

The plants were sown in a randomized block design, with two replicates at each location in each year. Each replication was designed based on a three-row plot that was 1 m long with a 26-cm row-to-row distance. The pot experiment was conducted in the same field in TA during 2011–2012; the pots were 28 cm in diameter and 23 cm deep, and they contained 14 kg of soil. Six plants were grown per pot, with two replications. During the growing seasons, damage from lodging, disease or pests did not occur.

### Trait measurements

Ten plants from the middle row were used to measure Sl, Ssn, Fsn, Gns, and Tkw at the mature stage in the field. In the pot experiment, all 12 plants grown in two pots (six plants per pot) were used to measure these traits at maturity. Data averages were used in the QTL analyses.

### Construction of the genetic linkage map

A previously constructed linkage map of the DH population with 323 markers located on 21 chromosomes was used (Zhang et al., 2008). (The markers included 284 simple sequence repeat (SSR) loci, 37 expressed sequence tag (EST) loci, 1 inter-SSR (ISSR) locus and 1 high molecular weight-glutenin subunit (HMW-GS) locus.) This linkage map covered a total length of 2485.7 cM, with an average distance of 7.67 cM between adjacent markers. The linked markers formed 24 linkage groups at LOD 4.0. The map was suitable for genome-wide QTL scanning in this study based on the recommended map distance for genome-wide QTL scanning of an interval length of 10 cM (Doerge, 2002).

### Data analyses

Statistical analyses (e.g., of normal distributions and correlations) were performed using the SPSS 13.0 statistical software package (SPSS, Stanford, Calif, USA) and Excel 2010.

ANOVA was performed using the PROC GLM procedure of SAS 8.0 (SAS Institute Inc., Cary, NC, USA). Heritability (h^2^) was calculated as h^2^ = $\sigma_{\text{g}}^{2}$/($\sigma_{\text{g}}^{2}+\sigma_{\text{ge}}^{2}$/r$+\sigma_{\varepsilon }^{2}$/re), where $\sigma_{\text{g}}^{2}$, $\sigma_{\text{ge}}^{2}$, and $\sigma_{\varepsilon }^{2}$ are estimates of genotype, genotype × environment and residual error variances, respectively.

QTL mapping was performed using the composite interval mapping method in IciMapping 4.0 (Wang, 2009; http://www.isbreeding.net) in individual environments. A walking speed of 1.0 cM and a logarithm of odds (LOD) score of 3.0 (based on 1000 permutations) were used to detect and declare the presence of a putative QTL.

### QTL nomenclature

To clarify the designations of the examined QTLs, the following rules were adopted: “Q” denotes “QTL”; the letter following “Q” is an abbreviation of its corresponding trait, which is followed by a dot and then the letters abbreviating Shandong Agricultural University; a number and an uppercase letter, “A,” “B,” or “D,” follow, which represent the chromosome number in a given wheat genome in which the corresponding QTL was detected; when a chromosome carries more than one QTL, a hyphen and serial number are added following the chromosome number and letter (e.g., *QFw.sdau-6A-2* denotes the second QTL for FW on chromosome 6A).

## Results

### Phenotypic data and correlation analyses

**Figure 1:**
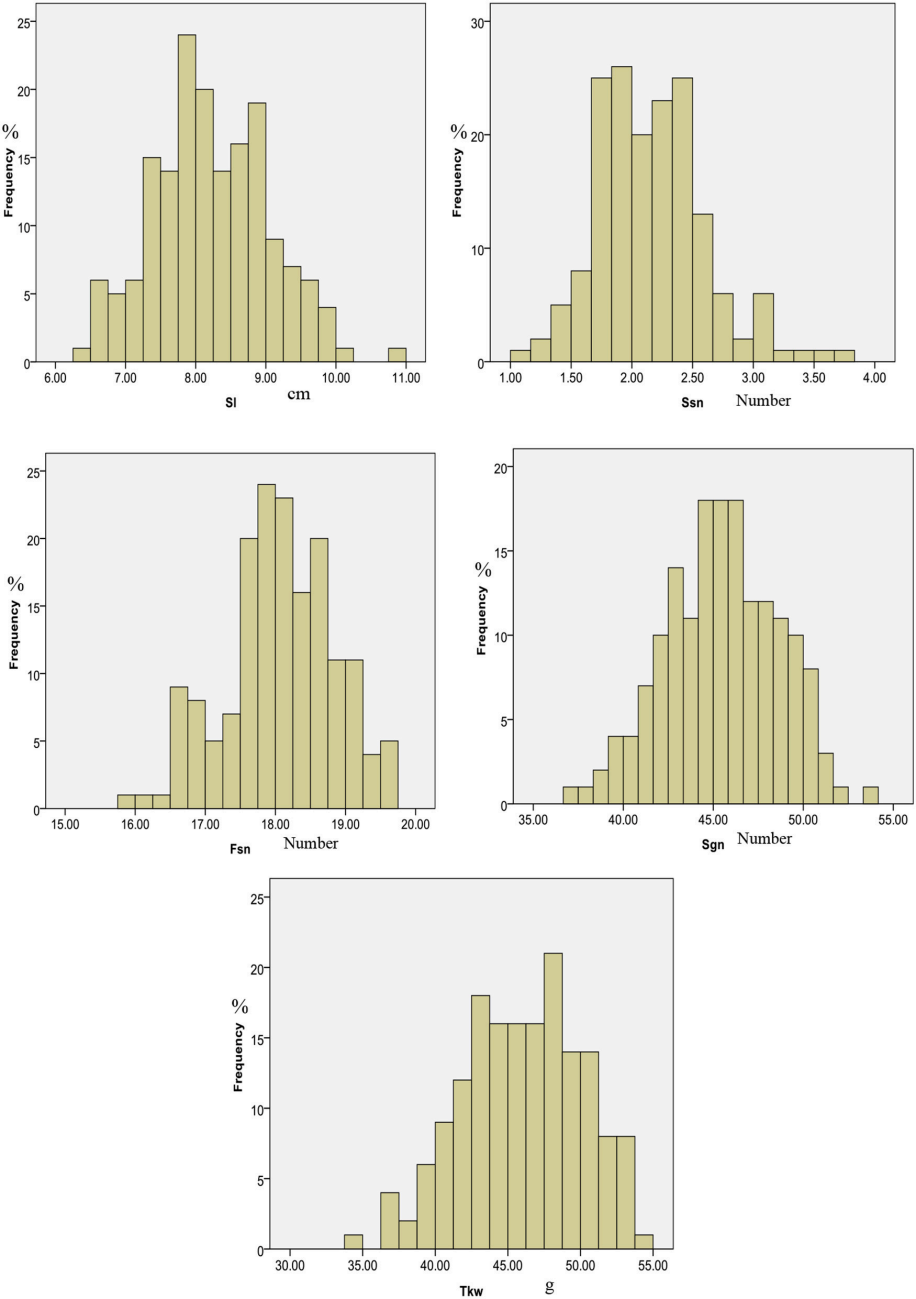
Distributions of five spike-related traits based on average data from four different treatments in two locations and 2 years.

Sl, Ssn and Fsn of the HP3 parent showed more variation than did those of YM57 for almost all treatments in both locations and years. In contrast, Gns of HP3 showed less variation than did that of YM57 for almost all treatments in both locations and years, except T2 and T3 in 2011TA (Table 1). In the DH population, the five traits exhibited approximately continuous variation in each treatment in the three environments (Table 1 and Figure S1). Transgressive segregation was observed in both the high and low sides in this population, indicating that alleles with positive effects were contributed by both parents. Additionally, the absolute values of skewness and kurtosis were almost < 1, indicating that the phenotypic data were approximately normally distributed in this population (Figure 1 and Figure S1).

**Table 1:** Distributions of five spike-related traits in parents and DH populations of wheat under different nitrogen supplying dates.

**Environment^a^**	**Treatment^b^**	**Parent**		**DH population**					
		**HP3**	**YM57**	**Mean**	**S.D**.	**Min**	**Max**	**Skewness**	**Kurtosis**
**Sl (cm)**									
2011JY	T0	7.81	7.22	8.03	0.90	5.87	10.71	0.06	−0.25
	T1	8.98	9.06	8.59	0.93	5.96	10.98	0.10	−0.15
	T2	9.10	8.80	8.79	0.95	6.68	11.50	0.18	−0.32
	T3	8.86	8.46	8.42	0.93	6.64	10.70	0.30	−0.39
2011TA	T0	8.34	7.90	7.93	0.94	6.08	12.62	0.70	2.85
	T1	8.68	8.24	8.44	0.98	5.76	12.10	0.14	0.55
	T2	8.60	8.46	8.44	1.02	6.36	12.56	0.44	0.64
	T3	8.46	8.14	8.41	0.95	6.40	11.84	0.25	0.22
2012TA	T0	8.10	7.53	7.72	1.04	5.33	11.23	0.42	0.27
	T1	7.63	7.37	7.71	0.96	5.70	10.63	0.32	0.33
	T2	8.56	7.90	8.74	1.01	5.70	11.46	0.04	0.09
	T3	7.50	7.50	7.55	0.91	5.50	10.83	0.44	0.45
**Ssn (no. per spike)**									
2011JY	T0	3.00	0.80	2.27	0.74	0.60	4.40	0.16	−0.20
	T1	2.20	1.20	2.40	0.83	0.80	5.00	0.78	0.73
	T2	2.20	1.20	2.40	0.83	0.80	5.00	0.78	0.73
	T3	2.40	1.60	2.43	0.83	0.80	6.60	1.20	3.71
2011TA	T0	1.60	1.20	1.40	0.69	0.00	4.40	0.95	1.84
	T1	2.40	1.00	1.92	0.50	0.70	3.40	0.22	0.33
	T2	2.00	0.60	1.75	0.46	0.80	3.20	0.16	0.03
	T3	2.40	1.70	1.88	0.47	0.60	3.00	0.00	−0.07
2012TA	T0	1.80	1.40	2.24	0.90	0.60	6.20	1.24	2.47
	T1	2.20	2.00	2.57	1.09	0.60	6.20	0.76	0.48
	T2	1.80	1.40	1.97	0.81	0.20	4.60	0.73	0.76
	T3	2.00	0.80	2.58	0.86	0.80	5.20	0.69	0.35
**Fsn (no. per spike)**									
2011JY	T0	20.20	18.40	17.99	1.25	14.40	20.80	−0.26	−0.33
	T1	20.20	19.40	18.48	1.16	15.60	21.40	−0.06	−0.49
	T2	20.20	18.60	18.01	1.24	13.40	20.60	−0.44	0.30
	T3	20.00	16.60	18.15	1.26	13.40	21.20	−0.53	1.14
2011TA	T0	17.20	17.20	17.04	1.07	14.20	19.60	−0.05	−0.29
	T1	16.60	17.00	16.90	0.77	14.70	18.90	−0.30	−0.04
	T2	17.80	17.40	17.03	0.85	15.20	19.20	0.18	−0.48
	T3	17.50	16.40	16.77	0.88	14.60	18.80	−0.10	−0.22
2012TA	T0	20.40	18.60	18.85	1.43	14.60	22.20	−0.20	−0.10
	T1	18.80	19.80	18.92	1.48	14.60	21.80	−0.31	0.01
	T2	20.20	18.60	19.75	1.37	15.40	22.60	−0.47	0.08
	T3	19.00	20.40	18.83	1.35	14.60	21.80	−0.40	0.03
**Gns (no. per spike)**									
2011JY	T0	45.80	50.00	42.41	5.78	30.00	58.20	0.36	−0.22
	T1	46.80	47.80	42.80	5.88	26.60	61.80	0.28	0.49
	T2	50.80	48.80	41.82	5.82	29.60	60.00	0.60	0.54
	T3	52.20	46.20	42.68	5.37	29.60	58.20	0.35	0.47
2011TA	T0	42.20	46.00	41.84	4.67	32.00	55.20	0.09	−0.46
	T1	44.70	50.70	45.02	4.14	33.60	57.10	−0.06	0.13
	T2	45.40	52.30	44.14	4.24	31.60	53.80	0.15	−0.04
	T3	40.90	45.10	43.54	3.75	34.70	53.60	0.03	−0.35
2012TA	T0	40.40	43.00	49.01	6.68	30.50	64.40	−0.07	−0.43
	T1	43.40	46.40	51.21	6.33	33.00	64.60	0.01	−0.22
	T2	45.40	45.40	52.80	5.93	39.40	64.60	−0.13	−0.71
	T3	43.40	49.80	48.18	6.03	35.00	62.60	0.14	−0.69
**Tkw (g)**									
2011JY	T0	44.0	46.0	46.54	4.57	28.6	55.5	−0.41	0.71
	T1	42.9	44.1	44.14	5.30	30.6	58.2	−0.14	−0.17
	T2	40.8	44.2	43.64	5.79	29.7	55.2	−0.11	−0.59
	T3	45.5	44.2	45.08	5.79	26.8	58.3	−0.30	−0.10
2011TA	T0	47.2	42.6	48.51	5.32	35.4	59.6	−0.41	−0.50
	T1	47.4	46.6	47.10	5.70	33.2	57.4	−0.29	−0.70
	T2	46.1	45.5	47.17	5.54	32.6	57.3	−0.29	−0.68
	T3	44.2	44.6	47.49	5.22	34.3	58.8	−0.33	−0.63
2012TA	T0	44.9	46.4	45.59	4.23	34.1	58.2	−0.02	0.08
	T1	46.5	43.4	45.36	3.82	33.3	55.0	−0.20	0.09
	T2	48.6	46.7	45.54	3.57	35.6	55.9	−0.07	0.38
	T3	50.8	46.8	45.22	3.89	35.0	54.7	0.07	−0.13

a *2011JY, Jiyuan in 2011; 2011TA, Tai'an in 2011; 2012TA, Tai'an in 2012*.

b *T0, no nitrogen fertilization treatment; T1, 120 kg/hm^2^pure nitrogen top-dressed fertilization at turning-green stage; T2, 120 kg/hm^2^pure nitrogen top-dressed fertilization at jointing stage; T3, 120 kg/hm^2^pure nitrogen top-dressed fertilization at the booting stage*.

Genetic variation of the DH population for the five traits was shown in Table 2, which presents the ANOVA results for the phenotypic data. ANOVA showed that genotype, environment and G × E had significant effects on Sl, Gns and Tkw. Genotype and environment had significant effects on Fsn. Ssn was significantly affected by environment. Heritability ranged from 0.37 to 0.97 (Table 2).

**Table 2:** Analysis of variance results and broad-sense heritabilities for spike traits in DH populations from two locations in 2 years.

**Trait**	**Mean squares**				**h^2^**
	**Genotype**	**Environment**	**G × E**	**Error**	
Sl	8.12^***^	108.81^***^	0.49^***^	0.19	0.97
Gns	119.32^***^	11564.84^***^	40.04^***^	23.98	0.92
Fsn	13.39^***^	791.98^***^	9.06	8.60	0.43
Ssn	14.51	138.15^***^	10.56	10.21	0.37
Tkw	198.26^***^	1290.99^***^	25.69^***^	4.20	0.95

*** *Significant at P < 0.001*.

Sl was significantly positively correlated with Ssn and Gns (Table 3), and Ssn was significantly negatively correlated with Fsn and Gns. Fsn and Tkw were also negatively correlated (Table 3). A significant positive correlation was observed between Ssn and Tkw and between Fsn and Gns.

**Table 3:** Correlation analyses among five spike-related traits using average data.

**CC^a^**	**Sl**	**Ssn**	**Fsn**	**Gns**	**Tkw**
Sl	1				
Ssn	0.244^**^	1			
Fsn	0.038	−0.199^**^	1		
Gns	0.160^*^	−0.418^**^	0.604^**^	1	
Tkw	−0.007	0.246^**^	−0.233^**^	−0.142	1

a CC, Correlation coefficient;

** Correlation is significant at the 0.01 level;

* *Correlation is significant at the 0.05 level*.

### Additive QTL analyses of five traits under nitrogen treatment

#### QTLs for Sl

**Figure 2:**
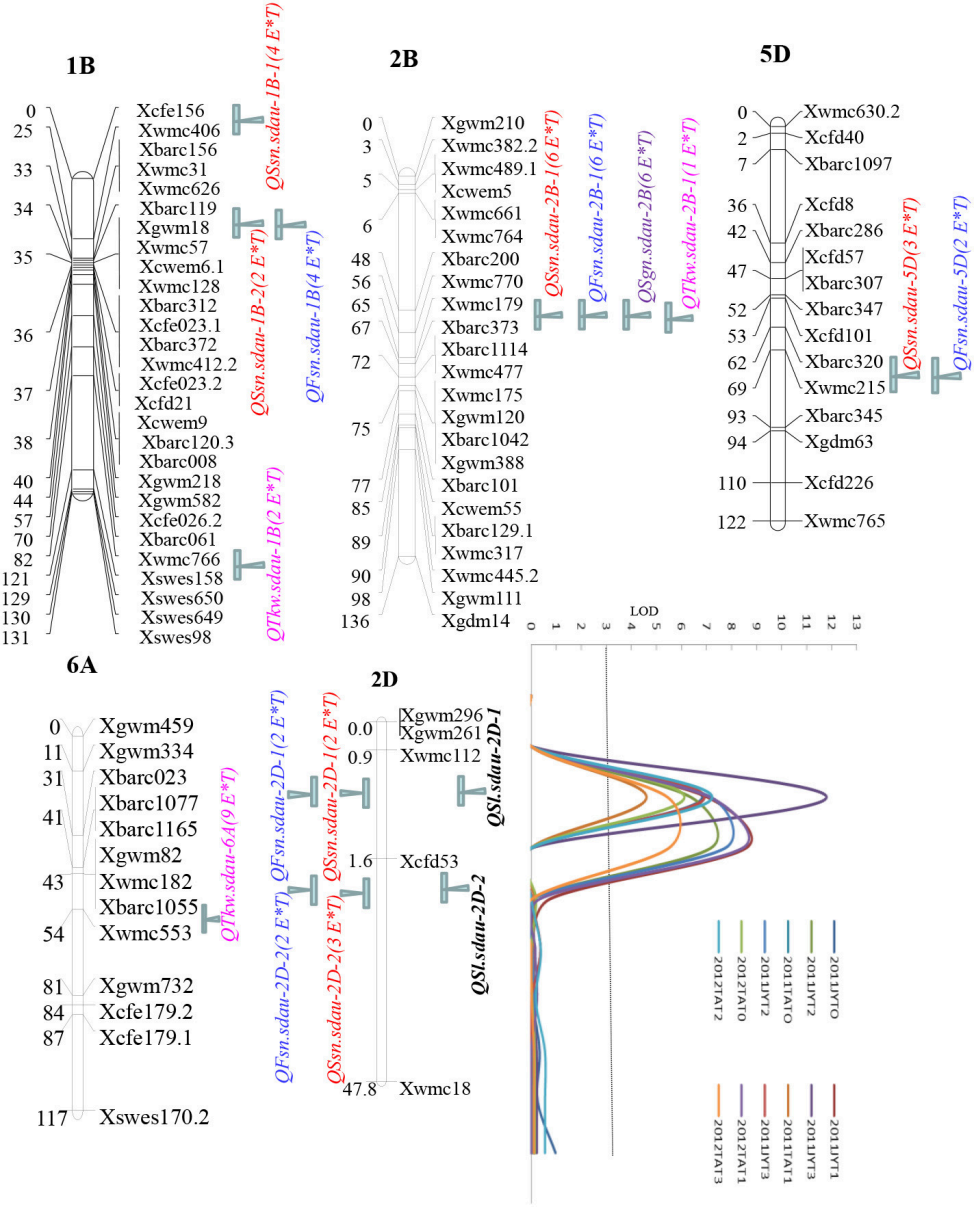
Linkage maps of important chromosome regions and co-localizing QTLs. LOD curves for QTLs were obtained from inclusive composite interval mapping (ICIM) of Sl under different nitrogen supply dates in the different locations and years. The horizontal dashed-dot line indicates the LOD threshold of 3.0 determined by permutations. E × T indicates environment × treatment. QTLs for Ssn, Fsn, Gns, Tkw, and Sl are shown in red, blue, purple, pink and black, respectively.

Eleven QTLs were detected on chromosomes 2A, 2D, 3A, 4B, 4D, 5D, 6B, and 7B under four different treatments in both locations and years (Table 4). These QTLs explained 5.19–24.2% of the phenotypic variation. In 2011JY, two major QTLs (*QSl.sdau-2D-1* and *QSl.sdau-2D-2*) were found on chromosome 2D, with close marker intervals *Xwmc112-Xcfd53-Xwmc18* (Table 4 and Figure 2). *QSl.sdau-2D-1* was detected under both T0 and T3, whereas *QSl.sdau-2D-2* was found under T1 and T2. The locations of these two QTLs were very similar and near marker *Xcfd53*. Additionally, the minor QTL *QSl.sdau-6B* was consistently detected under treatments T0, T2, and T3. In 2011TA under T0, T1 and T3, the stable major QTL *QSl.sdau-2D-1*, with flanking markers *Xwmc112* and *Xcfd53*, was found, whereas under T2, another major QTL, *QSl.sdau-2D-2*, was found (Table 4 and Figure 2). Notably, a minor QTL on chromosome 6B was consistently detected in three treatments (T0, T1, and T2). In 2012TA, two major QTLs, *QSl.sdau-2D-1*, and *QSl.sdau-2D-2*, were detected, with 14.64–24.2% of the phenotypic variation explained (PVE; Table 3 and Figure 2). *QSl.sdau-2D-1* was found under treatments T0 and T2, whereas *QSl.sdau-2D-2* was identified under T1 and T3. The minor QTL *QSl.sdau-6B* was detected only under the T1 treatment.

**Table 4:** Additive QTLs for spike length identified under different nitrogen supply dates in different years.

**Environment^a^**	**Treatment^b^**	**QTL**	**Position**	**Left marker**	**Right marker**	**LOD**	**PVE (%)**	**Add^c^**
2012 Tai'an	T0	*QSl.sdau-2D-1*	1	*Xwmc112*	*Xcfd53*	6.12	15.60	0.41
	T1	*QSl.sdau-2D-2*	3	*Xcfd53*	*Xwmc18*	8.45	24.20	0.45
	T1	*QSl.sdau-6B*	0	*Xcfa2187*	*Xgwm219*	3.41	6.88	0.25
	T2	*QSl.sdau-2D-1*	1	*Xwmc112*	*Xcfd53*	7.22	16.00	0.26
	T3	*QSl.sdau-2D-2*	3	*Xcfd53*	*Xwmc18*	5.50	14.64	0.34
2011 Jiyuan	T0	*QSl.sdau-2D-1*	1	*Xwmc112*	*Xcfd53*	6.83	15.88	0.41
	T0	*QSl.sdau-3A*	116	*Xwmc527*	*Xwmc264*	3.40	6.92	−0.24
	T0	*QSl.sdau-5D*	32	*Xbarc1097*	*Xcfd8*	3.32	6.32	0.23
	T0	*QSl.sdau-6B*	0	*Xcfa2187*	*Xgwm219*	3.16	5.19	0.21
	T1	*QSl.sdau-2D-2*	3	*Xcfd53*	*Xwmc18*	8.58	22.48	0.43
	T1	*QSl.sdau-7B*	12	*Xwmc273.1*	*Xcfd22.1*	3.66	7.57	0.25
	T2	*QSl.sdau-2D-2*	3	*Xcfd53*	*Xwmc18*	7.18	16.36	0.38
	T2	*QSl.sdau-4D*	20	*Xcfe188*	*Xbarc224*	3.15	6.65	−0.24
	T2	*QSl.sdau-6B*	0	*Xcfa2187*	*Xgwm219*	4.88	10.04	0.31
	T3	*QSl.sdau-2A*	68	*Xgwm558*	*Xbarc015*	3.961	6.84	−0.24
	T3	*QSl.sdau-2D-1*	1	*Xwmc112*	*Xcfd53*	11.79	22.89	0.44
	T3	*QSl.sdau-6B*	0	*Xcfa2187*	*Xgwm219*	3.18	5.37	0.22
2011 Tai'an	T0	*QSl.sdau-2D-2*	1	*Xwmc112*	*Xcfd53*	8.13	17.1	0.39
	T0	*QSl.sdau-6B*	0	*Xcfa2187*	*Xgwm219*	4.19	8.28	0.28
	T1	*QSl.sdau-2A*	68	*Xgwm558*	*Xbarc015*	3.15	6.91	−0.26
	T1	*QSl.sdau-2D-1*	1	*Xwmc112*	*Xcfd53*	4.60	10.39	0.32
	T1	*QSl.sdau-6B*	0	*Xcfa2187*	*Xgwm219*	3.20	6.86	0.27
	T2	*QSl.sdau-2D-2*	3	*Xcfd53*	*Xwmc18*	7.75	15.04	0.40
	T2	*QSl.sdau-2D-3*	205.1	*Xgdm93*	*Xwmc170.1*	3.75	6.56	−0.26
	T2	*QSl.sdau-6B*	0	*Xcfa2187*	*Xgwm219*	5.01	9.15	0.32
	T2	*QSl.sdau-7B*	12	*Xwmc273.1*	*Xcfd22.1*	5.35	9.34	0.31
	T3	*QSl.sdau-2D-1*	1	*Xwmc112*	*Xcfd53*	6.92	13.96	0.35
	T3	*QSl.sdau-4B*	17	*Xwmc657*	*Xwmc48*	3.37	6.87	0.25

a *2011JY, Jiyuan in 2011; 2011TA, Tai'an in 2011; 2012TA, Tai'an in 2012*.

b *T0, no nitrogen fertilization treatment; T1, 120 kg/hm^2^ pure nitrogen top-dressed fertilization at turning-green stage; T2, 120 kg/hm^2^ pure nitrogen top-dressed fertilization at jointing stage; T3, 120 kg/hm^2^ pure nitrogen top-dressed fertilization at the booting stage*.

c *Additive effect*.

In general, three stable QTLs were found on chromosomes 2D and 6B, of which two were major QTLs on chromosome 2D, with 10.38–24.2% PVE (Table 4 and Figure 2) in different environments. The marker interval *Xwmc112-Xcfd53-Xwmc18* was associated with these two major QTLs. Seven environments (2011JYT0, 2011JYT3, 2011TAT0, 2011TAT1, 2011TAT3, 2012TAT0, and 2012TAT2) were found to be involved in the major QTL *QSl.sdau-2D-1* located 1 cM between *Xwmc112* and *Xcfd53*, a location close to marker *Xwmc112*. Regarding the major QTL *QSl.sdau-2D-2*, the 2011JYT1, 2011JYT2, 2011TAT2, 2012TAT1, and 2012TAT3 environments were consistently identified. The location of this QTL at 3 cM is very close to marker *Xcfd53*, with a genetic distance of only 0.7 cM (Figure 2). Importantly, this locus was not affected by the different N top-dressed stage treatments, which indicated that this locus is very consistent. Therefore, these two markers (*Xwmc112* and *Xcfd53*) were selected for screening Sl in MAS breeding.

Additionally, the minor QTL *QSl.sdau-6B* was consistently detected in seven different treatments (2011JYT0, 2011JYT2, 2011JYT3, 2011TAT0, 2011TAT1, 2011TAT2, and 2012TAT1), with 5.19–10.39% PVE. *QSl.sdau-7B* was found in both 2011JYT1 and 2011TAT2, and *QSl.sdau-2A* was detected in 2011JYT3 and 2011TAT1. Therefore, in addition to the major locus, i.e., *QSl.sdau-6B*, these loci are important for improving Sl.

#### QTLs for Ssn

Thirteen QTLs for Ssn were found distributed on chromosomes 1B, 2B, 2D, 4A, 5A, 5D, and 7D under the four different treatments in both locations and years (Table 5). These QTLs explained from 5.14 to 20.3% of the phenotypic variation. In 2011JY, one stable QTL, *QSsn.sdau-2B-1*, was found in all four treatments, with 5.14–11.45% PVE. *QSsn.sdau-2D-2* was detected under both the T0 and T3 treatments, explaining 20.3% and 5.88% of the phenotypic variation, respectively, whereas *QSsn.sdau-1B-1* was found under T1 and T2, with 18.55 and 8.43% PVE, respectively. The remaining three QTLs were each identified in only one treatment. In 2011TA, *QSsn.sdau-2B-1* was detected under both the T0 and T2 treatments, whereas *QSsn.sdau-1B-1* was found under T1 and T2. Although *QSsn.sdau-2D-2*, with 15.24% PVE, and *QSsn.sdau-2D-1*, with 12.59% PVE, were each detected in only one treatment, the locations of these two major QTLs are very similar and near marker *Xcfd53*. Additionally, the minor QTL *QSsn.sdau-5D* was consistently detected under T1 and T3. In 2012TA, only two QTLs, *QSsn.sdau-5B*, and *QSsn.sdau-2D-1*, were detected, under treatment T1 and T3, respectively.

**Table 5:** Additive QTLs for sterile spikelet number identified under different nitrogen supply dates in different years.

**Environment^a^**	**Treatment^b^**	**QTL**	**Position**	**Left marker**	**Right marker**	**LOD**	**PVE (%)**	**Add^c^**
2011JY	T0	*QSsn.sdau-2B-1*	65	*Xwmc179*	*Xbarc373*	3.01	5.14	0.16
	T0	*QSsn.sdau-2D-2*	3	*Xcfd53*	*Xwmc18*	5.55	20.30	0.33
	T1	*QSsn.sdau-1B-1*	24	*Xcfe156*	*Xwmc406*	9.91	18.55	0.35
	T1	*QSsn.sdau-1B-2*	35	*Xbarc119*	*Xgwm18*	4.62	8.65	−0.24
	T1	*QSsn.sdau-2B-1*	65	*Xwmc179*	*Xbarc373*	6.53	11.45	0.27
	T1	*QSsn.sdau-4A*	44	*Xbarc078*	*Xwmc722*	3.24	5.57	0.19
	T2	*QSsn.sdau-1B-1*	24	*Xcfe156*	*Xwmc406*	5.30	8.43	0.30
	T2	*QSsn.sdau-2B-1*	65	*Xwmc179*	*Xbarc373*	3.54	5.40	0.24
	T2	*QSsn.sdau-5A*	108	*Xcfe223*	*Xwmc273.3*	4.36	7.94	0.29
	T2	*QSsn.sdau-5D*	69	*Xbarc320*	*Xwmc215*	10.09	17.65	−0.44
	T3	*QSsn.sdau-2B-1*	65	*Xwmc179*	*Xbarc373*	3.07	6.19	0.20
	T3	*QSsn.sdau-2D-2*	3	*Xcfd53*	*Xwmc18*	3.02	5.88	0.20
2011TA	T0	*QSsn.sdau-1B-2*	35	*Xbarc119*	*Xgwm18*	3.37	7.39	0.19
	T0	*QSsn.sdau-2B-1*	65	*Xwmc179*	*Xbarc373*	3.23	7.03	0.18
	T0	*QSsn.sdau-7D*	130	*Xwmc630.1*	*Xgdm67*	3.32	8.49	0.21
	T1	*QSsn.sdau-1B-1*	24	*Xcfe156*	*Xwmc406*	3.75	6.62	0.13
	T1	*QSsn.sdau-4A*	44	*Xbarc078*	*Xwmc722*	3.14	5.23	0.12
	T1	*QSsn.sdau-5B.2*	24	*Xbarc232*	*Xwmc235*	4.09	6.76	−0.13
	T1	*QSsn.sdau-5D*	69	*Xbarc320*	*Xwmc215*	3.25	5.30	−0.12
	T2	*QSsn.sdau-1B-1*	24	*Xwmc406*	*Xbarc156*	3.72	6.57	0.12
	T2	*QSsn.sdau-2B-1*	65	*Xwmc179*	*Xbarc373*	3.06	6.22	0.11
	T2	*QSsn.sdau-2D-1*	1	*Xwmc112*	*Xcfd53*	5.72	12.59	0.16
	T3	*QSsn.sdau-2B-2*	75	*Xbarc1042*	*Xgwm388*	3.18	6.31	0.12
	T3	*QSsn.sdau-2D-2*	3	*Xcfd53*	*Xwmc18*	4.86	15.24	0.18
	T3	*QSsn.sdau-5D*	69	*Xbarc320*	*Xwmc215*	3.99	9.59	−0.15
2012TA	T1	*QSsn.sdau-5B*	2	*Xbarc1125*	*Xgwm213*	4.07	9.39	0.33
	T3	*QSsn.sdau-2D-1*	1	*Xwmc112*	*Xcfd53*	3.43	8.09	0.24

a and b *are the same as those for Table 3*.

c *Epistatic effect*.

In general, four stable QTLs were found on chromosomes 1B, 2B, 2D, and 5D in more than three environments, with 18.55, 11.45, 17.65, and 15.25% of the maximum PVE, respectively.

#### QTLs for Fsn

Twenty QTLs for Fsn were detected, involving 13 chromosomes (1B, 2B, 2D, 3A, 3B, 3D, 4B, 5A, 5B, 5D, 6A, 7A, and 7D) under the four different treatments in both locations and years (Table 6). These QTLs explained from 4.37 to 34.74% of the phenotypic variation. In 2011JY, one stable QTL, *QFsn.sdau-2B-1*, was detected under all four treatments, with 6.18–28.43% PVE. *QFsn.sdau-5D* was found under T1 and T3, with 16.8 and 8.55% PVE, respectively. Additionally, four other major QTLs, *QFsn.sdau-1B, QFsn.sdau-2D-2, QFsn.sdau-5D*, and *QFsn.sdau-3D*, were each identified in only one treatment. In 2011TA, *QFsn.sdau-1B* was found in T0, T2, and T3, with 34.73, 6.39, and 14.74% PVE, respectively. *QFsn.sdau-2B-1* was detected in treatments T0 and T2. *QFsn.sdau-2D-1, QFsn.sdau-2D-2*, and *QFsn.sdau-2D-3* were each found in only one treatment. In 2012TA, *QFsn.sdau-6A-1* was detected in T0 and T1, with 11.47 and 9.2% PVE, respectively. *QFsn.sdau-2D-1* and *QFsn.sdau-2D-3* were each found in only one treatment.

**Table 6:** Additive QTLs for fertile spikelet number identified under different nitrogen supply dates in different years.

**Environment^a^**	**Treatment^b^**	**QTL**	**Position**	**Left marker**	**Right marker**	**LOD**	**PVE (%)**	**Add^c^**
2011JY	T0	*QFsn.sdau-1B*	35	*Xbarc119*	*Xgwm18*	14.14	28.24	−0.67
	T0	*QFsn.sdau-2B-1*	65	*Xwmc179373*	*Xbarc373*	8.42	15.84	−0.49
	T0	*QFsn.sdau-2D-2*	3	*Xcfd53*	*Xwmc18*	5.72	10.15	−0.39
	T0	*QFsn.sdau-3B*	53	*Xgwm144*	*Xgwm299*	3.26	5.68	0.32
	T0	*QFsn.sdau-3D*	9	*Xbarc376*	*Xgdm72*	3.17	6.00	0.30
	T0	*QFsn.sdau-5B*	2	*Xbarc1125*	*Xgwm213*	3.02	5.10	0.28
	T1	*QFsn.sdau-2B-1*	65	*Xwmc179*	*Xbarc373*	3.01	6.18	−0.28
	T1	*QFsn.sdau-5D*	69	*Xbarc320*	*Xwmc215*	7.13	16.80	−0.47
	T2	*QFsn.sdau-2B-1*	65	*Xwmc179*	*Xbarc373*	13.90	28.43	−0.65
	T2	*QFsn.sdau-2B-2*	75	*Xbarc1042*	*Xgwm388*	8.39	15.57	0.49
	T2	*QFsn.sdau-3D*	9	*X barc376*	*X gdm72*	5.86	10.91	0.41
	T2	*QFsn.sdau-5A.2*	13	*Xcwem32.2*	*Xwmc59*	3.41	5.84	−0.30
	T2	*QFsn.sdau-6A-2*	84	*Xcfe179.2*	*Xcfe179.1*	3.10	5.23	−0.28
	T2	*QFsn.sdau-7D-2*	163	*Xwmc634*	*Xwmc273.2*	4.00	7.12	−0.33
	T3	*QFsn.sdau-2B-1*	65	*Xwmc179*	*Xbarc373*	3.35	6.63	−0.32
	T3	*QFsn.sdau-5D*	69	*Xbarc320*	*Xwmc215*	3.63	8.55	−0.36
2011TA	T0	*QFsn.sdau-1B*	35	*Xbarc119*	*Xgwm18*	13.12	34.74	−0.64
	T0	*QFsn.sdau-2B-1*	65	*Xwmc179*	*Xbarc373*	3.21	6.57	−0.27
	T2	*QFsn.sdau-1B*	35	*Xbarc119*	*Xgwm18*	4.33	6.39	0.23
	T2	*QFsn.sdau-2B-1*	65	*Xwmc179*	*Xbarc373*	3.00	4.37	−0.18
	T2	*QFsn.sdau-2D-1*	1	*Xwmc112*	*Xcfd53*	4.87	7.29	−0.23
	T2	*QFsn.sdau-2D-4*	102	*Xbarc349.1*	*Xcfd161*	4.50	8.20	0.23
	T2	*QFsn.sdau-4B*	0	*Xwmc125*	*Xwmc47*	4.44	6.52	0.22
	T2	*QFsn.sdau-5B.2*	0	*Xbarc36*	*Xbarc140*	3.36	4.86	−0.19
	T2	*QFsn.sdau-6D*	0	*Xwmc412.1*	*Xcfd49*	6.97	10.61	−0.28
	T2	*QFsn.sdau-7A*	6	*Xwmc593*	*Xbarc157.2*	3.17	4.66	0.18
	T3	*QFsn.sdau-1B*	35	*Xbarc119*	*Xgwm18*	7.58	14.74	0.34
	T3	*QFsn.sdau-2D-2*	3	*Xcfd53*	*Xwmc18*	4.00	7.68	−0.24
	T3	*QFsn.sdau-2D-3*	53	*Xwmc18*	*Xwmc170.2*	5.04	9.08	0.26
	T3	*QFsn.sdau-7D-1*	115	*Xgwm676*	*Xgwm437*	4.36	7.66	0.25
2012TA	T0	*QFsn.sdau-6A-1*	36	*Xbarc023*	*Xbarc1077*	3.57	11.47	−0.48
	T1	*QFsn.sdau-3A*	126	*Xwmc264*	*Xcfa2193*	3.97	8.83	0.44
	T1	*QFsn.sdau-6A-1*	36	*Xbarc023*	*Xbarc1077*	3.25	9.21	−0.45
	T2	*QFsn.sdau-2D-1*	1	*Xwmc112*	*Xcfd53*	4.48	9.85	−0.43
	T2	*QFsn.sdau-2D-3*	53	*Xwmc18*	*Xwmc170.2*	3.01	6.66	0.35

a and b *are the same as those for Table 3*.

c *Epistatic effect*.

Overall, two stable QTLs were detected on chromosomes 1B and 2B in more than three environments. Three QTLs on chromosome 2D (*QFsn.sdau-2D-1, QFsn.sdau-2D-2* and *QFsn.sdau-2D-3*) were found in two environments, involving the marker interval *Xwmc112*-*Xcfd53*-*Xwmc18*.

#### QTLs for Gns

Eight QTLs for Gns were detected on chromosomes 1A, 2B, 2D, 3B, 3D, 4A, and 7A in all environments, with 6.74–17.24% PVE (Table 7). In 2011JY, two QTLs, *QGns.sdau-4A-2* and *QGns.sdau-2B*, were identified in T1 and T3, with 6.77 and 13.77% PVE, respectively. In 2011TA, *QGns.sdau-2B* was consistently detected in treatments T0, T2 and T3, with 17.15, 17.24, and 9.36% PVE, respectively. One major QTL, *QGns.sdau-4A-1*, was found in the T1 treatment. In 2012TA, *QGns.sdau-2B* was found in both T0 and T1, and the other five QTLs were each identified in only one treatment. In general, one stable QTL, *QGns.sdau-2B*, was detected in more than three environments.

**Table 7:** Additive QTLs for spike grain number identified under different nitrogen supply dates in different years.

**Environment^a^**	**Treatment^b^**	**QTL**	**Position**	**Left marker**	**Right marker**	**LOD**	**PVE (%)**	**Add^c^**
2011JY	T1	*QGns.sdau-4A-2*	35	*Xwmc497*	*Xwmc219*	3.29	6.77	−1.50
	T3	*QGns.sdau-2B*	65	*Xwmc179*	*Xbarc373*	5.56	13.77	−1.94
2011TA	T0	*QGns.sdau-2B*	65	*Xwmc179*	*Xbarc373*	6.22	17.15	−1.92
	T1	*QGns.sdau-4A-1*	5	*Xwmc718*	*Xwmc262*	4.72	10.57	−1.34
	T2	*QGns.sdau-2B*	65	*Xwmc179*	*Xbarc373*	7.24	17.24	−1.74
	T3	*QGns.sdau-2B*	65	*Xwmc179*	*Xbarc373*	4.78	9.36	−1.14
	T3	*QGns.sdau-2D*	75	*Xbarc349.2*	*Xbarc349.1*	3.28	7.46	1.04
2012TA	T0	*QGns.sdau-2B*	65	*Xwmc179*	*Xbarc373*	3.2	7.15	−1.79
	T0	*QGns.sdau-4A-1*	5	*Xwmc718*	*Xwmc262*	4.75	11.24	−2.24
	T1	*QGns.sdau-1A*	54	*Xcfd59*	*Xwmc402.2*	5.14	9.83	2.00
	T1	*QGns.sdau-2B*	65	*Xwmc179*	*Xbarc373*	3.92	7.42	−1.71
	T1	*QGns.sdau-3B*	66	*Xgwm566*	*Xcfe009*	3.49	6.75	1.67
	T1	*QGns.sdau-3D*	78	*Xcfd223*	*Xbarc323*	3.22	7.53	1.73
	T1	*QGns.sdau-7A*	78	*Xwmc530*	*Xcfa2123*	3.74	7.22	1.70

a and b *are the same as those for Table 3*.

c *Epistatic effect*.

#### QTLs for Tkw

Eighteen QTLs for Tkw were identified on chromosomes 1A, 1B, 2B, 2D, 3A, 4B, 4D, 5B, 6A, 6D, and 7D in all environments, with 4.13–15.18% PVE (Table 8). In 2011JY, *QTkw.sdau-6A* was consistently detected in all four treatments, with 15.18% of the highest PVE, whereas *QTkw.sdau-3A-1* and *QTkw.sdau-5B* were found in three treatments. In 2011TA, *QTkw.sdau-6A* was consistently detected in all four treatments, with 11.41, 7.04, 14.65, and 12.09% PVE. One major QTL, *QTkw.sdau-2B-1*, was identified in only the T0 treatment, with 15.93% PVE. In 2012TA, one major stable QTL, *QTkw.sdau-1B*, was detected in T0 and T2, explaining 21.03 and 10.01%, respectively, of the phenotypic variation. *QTkw.sdau-4B* and *QTkw.sdau-6D* were each identified in only one treatment, with 19.18 and 10.18% PVE, respectively. *QTkw.sdau-6A* was detected in the T2 treatment.

**Table 8:** Additive QTLs for thousand-kernel weight identified under different nitrogen supplying dates in different years.

**Environment^a^**	**Treatment^b^**	**QTL**	**Position**	**Left marker**	**Right marker**	**LOD**	**PVE (%)**	**Add^c^**
2011JY	T0	*QTkw.sdau-3A-1*	146	*Xwmc264*	*Xcfa2193*	3.87	10.57	1.47
	T0	*QTkw.sdau-6A*	43	*Xbarc1055*	*Xwmc553*	5.11	10.88	1.50
	T1	*QTkw.sdau-3A-1*	146	*Xwmc264*	*Xcfa2193*	3.61	8.75	1.56
	T1	*QTkw.sdau-5B*	68	*Xgwm213*	*Xswes861.2*	3.30	6.43	−1.39
	T1	*QTkw.sdau-6A*	43	*Xbarc1055*	*Xwmc553*	3.92	7.80	1.48
	T2	*QTkw.sdau-5B*	68	*Xgwm213*	*Xswes861.2*	3.16	6.03	−1.46
	T2	*QTkw.sdau-6A*	43	*Xbarc1055*	*Xwmc553*	3.01	5.87	1.39
	T3	*QTkw.sdau-3A-1*	146	*Xwmc264*	*Xcfa2193*	5.82	8.99	1.71
	T3	*QTkw.sdau-4D*	0	*Xbarc334*	*Xwmc331*	3.23	4.71	1.24
	T3	*QTkw.sdau-5B*	68	*Xgwm213*	*Xswes861.2*	3.01	4.13	−1.21
	T3	*QTkw.sdau-6A*	43	*Xbarc1055*	*Xwmc553*	9.35	15.18	2.24
	T3	*QTkw.sdau-7D-1*	123	*Xgwm676*	*Xgwm437*	4.10	7.00	1.56
	T3	*QTkw.sdau-7D-2*	162	*Xgdm67*	*Xwmc634*	5.87	9.18	−1.73
2011TA	T0	*QTkw.sdau-2B-1*	65	*Xwmc179*	*Xbarc373*	8.77	15.93	2.08
	T0	*QTkw.sdau-2B-2*	75	*Xbarc1042*	*Xgwm388*	3.25	5.66	−1.24
	T0	*QTkw.sdau-2D*	67	*Xwmc170.2*	*Xgwm539*	4.92	8.61	1.54
	T0	*QTkw.sdau-6A*	43	*Xbarc1055*	*Xwmc553*	6.04	11.41	1.77
	T1	*QTkw.sdau-1A*	55	*Xcfd59*	*Xwmc402.2*	3.16	4.44	1.21
	T1	*QTkw.sdau-3A-2*	177	*Xbarc51*	*Xbarc157.1*	3.65	5.23	1.28
	T1	*QTkw.sdau-4D*	0	*Xbarc334*	*Xwmc331*	3.77	5.27	1.29
	T1	*QTkw.sdau-5B.2*	20	*Xbarc140*	*Xbarc142*	5.47	7.80	−1.57
	T1	*QTkw.sdau-6A*	43	*Xbarc1055*	*Xwmc553*	4.19	7.04	1.50
	T1	*QTkw.sdau-6D*	56	*Xcfd13*	*Xbarc054*	5.68	8.85	−1.68
	T2	*QTkw.sdau-6A*	43	*Xbarc1055*	*Xwmc553*	5.86	14.64	2.10
	T3	*QTkw.sdau-2D*	67	*Xwmc170.2*	*Xgwm539*	3.1035	6.82	1.36
	T3	*QTkw.sdau-6A*	45	*Xbarc1055*	*Xwmc553*	4.98	12.09	1.81
2012TA	T0	*QTkw.sdau-1B*	101	*Xwmc766*	*Xswes158*	7.09	21.03	1.91
	T1	*QTkw.sdau-4B*	8	*Xwmc413*	*Xcfd39.2*	9.93	19.18	1.63
	T1	*QTkw.sdau-5B.2*	20	*Xbarc140*	*Xbarc142*	3.21	5.56	−0.88
	T1	*QTkw.sdau-6D*	56	*Xcfd13*	*Xbarc054*	4.85	10.18	−1.19
	T2	*QTkw.sdau-1B*	101	*Xwmc766*	*Xswes158*	3.10	10.01	1.05
	T2	*QTkw.sdau-2B-3*	88	*Xcwem55*	*Xbarc129.1*	3.85	8.51	−1.02
	T2	*QTkw.sdau-6A*	43	*Xbarc1055*	*Xwmc553*	3.63	8.13	1.00

a and b *are the same as those for Table 3*.

c *Epistatic effect*.

Therefore, three stable QTLs, *QTkw.sdau-6A, QTkw.sdau-3A-1* and *QTkw.sdau-5B*, were identified in more than three environments.

#### Epistatic QTL analysis of five traits

A total of 10 major epistatic QTLs were detected, with 11.44–33.78% PVE, for the five spike-related traits (Table 9). Two, one, three, one and three QTLs were identified for Sl, Ssn, Fsn, Gns, and Tkw, respectively, but only in one environment.

**Table 9:** Epistatic QTLs identified for spike-related traits under different nitrogen supply dates in different years.

**Environment^a^**	**Treatment^b^**	**QTL_1**	**Position 1**	**Marker interval**	**QTL_2**	**Position 2**	**Marker interval**	**LOD**	**PVE (%)**	Add^*^Add^c^
**Sl**										
2011TA	T1	*QSl.sdau-2A*	55	*Xwmc177-Xgwm558*	*QSl.sdau-7A*	55	*Xbarc259-Xwmc596*	5.32	11.88	−0.34
	T3	*QSl.sdau-1A*	30	*Xwmc728.1-Xwmc550*	*QSl.sdau-3A*	55	*Xswes107-Xbarc86*	5.07	15.94	0.41
Ssn										
2012TA	T0	*QSsn.sdau-1A*	35	*Xwmc728.1- wmc550*	*QSsn.sdau-6D*	150	*Xswes679.1- cfa2129*	5.01	33.78	0.67
Fsn										
2011JY	T1	*QFsn.sdau-4D*	175	*Xbarc237- Xcfe254*	*QFsn.sdau-6A*	35	*Xbarc023- Xbarc1077*	5.66	18.92	−0.52
2012TA	T0	*QFsn.sdau-2D*	75	*Xbarc349.2- Xbarc349.1*	*QFsn.sdau-3A*	100	*Xwmc489.3- Xcfa2134*	5.24	15.00	0.56
	T1	*QFsn.sdau-1B*	55	*Xgwm582- Xcfe026.2*	*QFsn.sdau-5B.2*	0	*Xbarc36- Xbarc140*	5.72	11.44	0.52
**Gns**										
2011JY	T0	*QGns.sdau-1A*	65	*Xgwm498- Xcwem6.2*	*QGns.sdau-7D*	195	*Xwmc14- Xwmc42*	5.31	20.97	3.02
Tkw										
2011JY	T2	*QTkw.sdau-3A*	60	*Xswes107- Xbarc86*	*QTkw.sdau-5B*	0	*Xgwm133.1- Xwmc73*	5.84	16.94	2.32
	T2	*QTkw.sdau-7A*	5	*Xwmc593- Xbarc157.2*	*QTkw.sdau-7D*	150	*Xgdm67- Xwmc634*	5.38	11.95	−2.00
	T3	*QTkw.sdau-1A*	0	*Xgwm259- Xcwem32.1*	*QTkw.sdau-7D*	55	*Xbarc244- Xbarc352*	5.76	13.25	2.17

a and b *are the same as those for Table 3*.

c *Epistatic effect*.

Actually, those major stable QTLs which were not affected by the nitrogen fertilization treatments or years or both should be paid more attention in this study. By comparison, 16 stable QTLs were found for five traits, including *QSl.sdau-2D-1, QSl.sdau-2D-2, QSl.sdau-6B, QSsn.sdau-2B-1, QSsn.sdau-2D-2, QSsn.sdau-1B-1, QFsn.sdau-2B-1, QFsn.sdau-1B, QFsn.sdau-6A-1, QFsn.sdau-5D, QFsn.sdau-3D, QGns.sdau-2B, QTkw.sdau-3A-1, QTkw.sdau-6A, QTkw.sdau-1B*, and *QTkw.sdau-5B*. Of these 16 QTLs, only the *QSl.sdau-2D-1*, and *QSl.sdau-2D-2* for Sl were major QTLs almost in all environments, and the remained QTLs showed major only in one environment or minor in all environments. The QTLs newly identified in T1, T2 or T3, but not detected in T0, could be induced expression by nitrogen fertilization, such as *QSl.sdau-7B, QSl.sdau-4D, QSl.sdau-2A, QSsn.sdau-4A, QSsn.sdau-5A, QFsn.sdau-7D-2*, etc. These QTLs perhaps were affected by nitrogen level.

#### Validation of the two flanking markers of QTLs for Sl

Because the identified major QTLs for Sl were stable in different environments, the two flanking markers *Xwmc112* and *Xcfd53* were validated using different varieties and populations.

**Figure 3:**
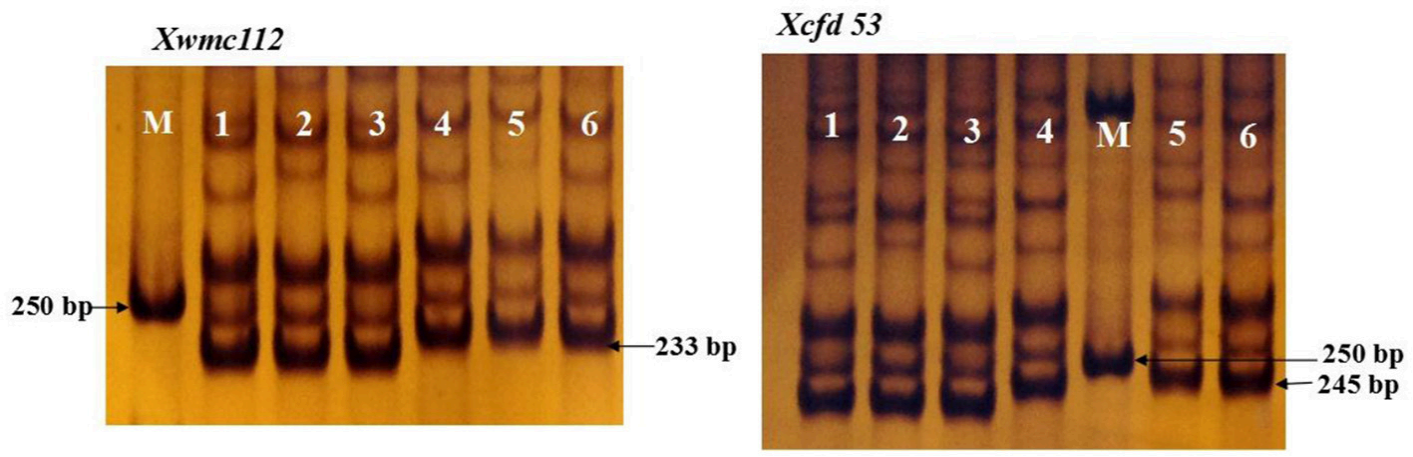
The size of PCR fragments of *Xcfd53* and *Xwmc112* in six varieties. M: DL2000; lanes 1, 2, and 3 represent the short-spike varieties SN19, SN20, and SN8355; and lanes 4, 5, and 6 represent the long-spike varieties *Elytrigia elongata*, SN62008 and SN08-29.

First, six different cultivars were used to evaluate these two markers. The sizes of the PCR fragments were 233 and 245 bp in the materials with long spikes, such as *Elytrigia elongata*, SN62008 and SN08-29 (Figure 3).

**Figure 4:**
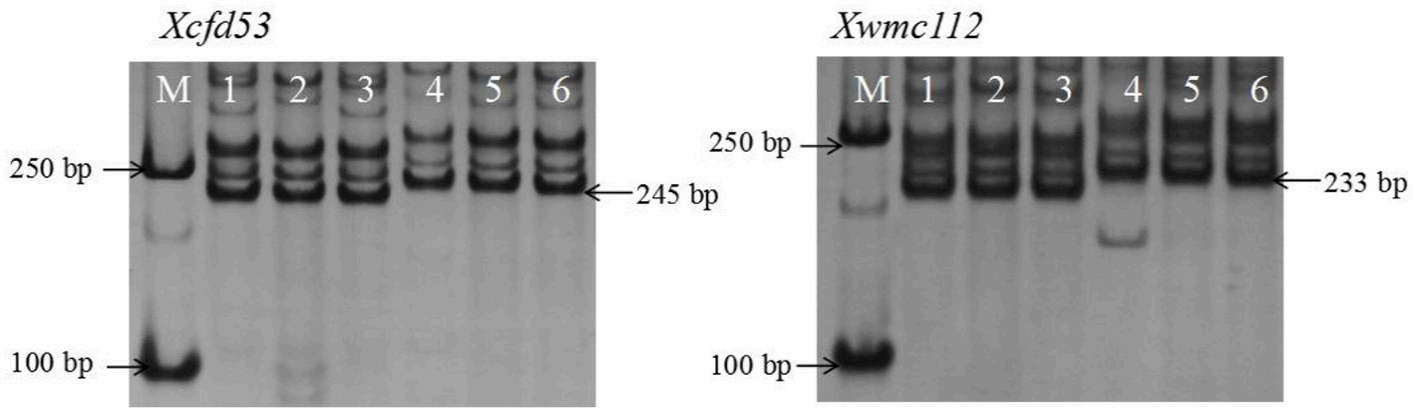
The size of PCR fragments of *Xcfd53* and *Xwmc112* in six extreme individuals of the BC_3_F_2_ population. M: DL2000; lanes 1, 2, and 3 represent short-spike materials; and lanes 4, 5, and 6 represent long-spike materials.

Then, these markers were used to evaluate the BC_3_F_2_ population derived from *Elytrigia elongata* (donor parent) and common wheat SN20 (recurrent parent). Sl was classified into four categories: long, ≥11 cm; medium-long, 9.1–11 cm; medium, 7.1–9 cm; and short, ≤ 7.0 cm. Then, extreme individuals (approximately 60) were used to evaluate the markers. After electrophoresis, fragments of 245 and 233 bp were detected in the individuals with extremely long spikes but not in those with extremely short spikes (Figure 4). These findings indicated that these two markers could be used to select for Sl in MAS.

## Discussion

### Some critical chromosome regions and co-localizing QTLs

A few previous studies involving spike-related traits have reported certain QTL clusters (Jantasuriyarat et al., 2004; Kumar et al., 2007; Ma et al., 2007; Chu et al., 2008; Wang et al., 2011; Cui et al., 2012; Yang et al., 2012; Islamovic et al., 2013; Liu et al., 2016; Zhai et al., 2016). In this study, 70 QTLs for five spike-related traits were detected, 15 of which formed eight clusters on five chromosomes (1B, 2B, 2D, 5D, and 6A) (Figure 2). Three important regions were detected on chromosome 1B, with four stable QTLs controlling Ssn, Fsn, and Tkw. A fourth primary region with four stable QTLs influencing Ssn, Fsn, Gns and Tkw was delineated between markers *Xwmc179* and *Xbarc373* on chromosome 2B. With regard to chromosome 2D, two important regions were found in the *Xwmc112*-*Xcfd53*-*Xwmc18* interval, with six stable QTLs related to Sl, Ssn, and Fsn. The marker interval *Xbarc320*-*Xwmc215* on chromosome 5D was found to harbor two stable QTLs controlling Ssn and Fsn. The remaining critical region with a consistent QTL was the marker interval *Xbarc1055*-*Xwmc553* on chromosome 6A, involving Tkw. These QTLs were stable in most environments. Co-localizing QTLs for different spike-related traits on many wheat chromosomes, such as 2D, 4A, 4B, 5A, 5B, 6A, and 7B, have been reported (Ma et al., 2007; Cui et al., 2012; Xu et al., 2014; Liu et al., 2016; Zhai et al., 2016).

In this study, the co-localizing QTLs primarily mapped to chromosomes 1B, 2B, 2D, and 5D. These co-localizing QTLs were related to Ssn and Fsn (chromosomes 1B and 5D); Ssn, Fsn, Gns, and Tkw (chromosome 2B); and Sl, Ssn and Fsn (chromosome 2D), particularly Ssn and Fsn (1B, 2B, 2D, and 5D). Therefore, Gns and Tkw can be expected to be improved by enhancing Sl and Ssn, respectively. The markers around these QTLs might be useful for marker-assisted breeding. Co-localizing QTLs for Ssn and Fsn were found on four chromosomes, with a significantly negative correlation coefficient between them, indicating that the loci controlling these two traits are typically dependent and that increasing Fsn would likely, to some extent, result in a decrease in Ssn, which is consistent with practical breeding experience.

### Comparison of the present findings with previous research

By affecting the number of spikelets, Fsn, and Gns, Sl is an important indirect factor that influences wheat yield, indicating that breeders should focus more attention on this phenotype (Ijaz and Kashif, 2013). Previous studies on QTL mapping for Sl using different populations such as RIL, IF2, ITMI RIL, F_2:3_, and DH populations have detected many major QTLs involving eleven chromosomes (1A, 1B, 2B, 2D, 4A, 4D, 5A, 5B, 6A, 6B, 7A, and 7D) (Jantasuriyarat et al., 2004; Kumar et al., 2007; Ma et al., 2007; Chu et al., 2008; Yang et al., 2012; Islamovic et al., 2013). In this study, QTLs for Sl were similarly detected on chromosomes 2B, 2D, 6B, and 7A, but the consistent major QTL was only detected on chromosome 2D, with 24.2% of the highest PVE. Ma et al. (2007) detected one major QTL, *QSpl.nau-2D*, with 20% PVE, and Kumar et al. (2007) and Wang et al. (2011) each found one major QTL on chromosome 2D, with 11.36 and 13.61% PVE, respectively. In the present study, a stable minor QTL on chromosome 6B was identified along with two major epistatic QTLs. These results indicate that Sl is primarily controlled by a major QTL/gene but is also affected by a minor QTL/gene as well as interactions between QTLs/genes. In 2014, the near-isogenic line (NIL) *QSpl.nau-2D* was developed by MAS using Mianyang 99–323 as the recurrent parent to validate its genetic effect and determine its precise location (Wu et al., 2014). *QSpl.nau-2D* was designated HL1 because it functions as a single gene and conditions Sl in a partially dominant manner. HL1 was subsequently precisely mapped to the interval flanked by *Xcfd53* and *DG371*, at 0.9 cM, resulting in longer spikes and a higher grain weight (Wu et al., 2014).

The common flanking marker “*Xcfd53*” of the major QTL was detected in the present research, and the genetic distance between *Xwmc112* and *Xcfd53* was found to be 0.7 cM, which is smaller than that between *Xcfd53* and *DG371*. These results demonstrate that the major QTL found on chromosome 2D in this research is valid and important for improving Sl. Importantly, the different environments and N top-dressed stage treatments did not affect this major QTL. Xu et al. (2014) also identified the stable QTL (*QSl-2D*) in the same marker interval (*Xwmc112* and *Xcfd53*), which was also not influenced by nitrogen and phosphorus fertilization. This QTL in our research is very consistent and has been used in molecular breeding. The size of amplification fragments for *Xwmc112* and *Xcfd53* using wheat samples with a long Sl was 233 and 245 bp, respectively. Wu et al. (2014) also found that an NIL with the *Xcfd53*-*DG371* interval had a long Sl. Additionally, one consistent minor QTL on chromosome 6B was identified in the present research.

Only a few studies to date have simultaneously reported QTLs for Ssn and Fsn (Li et al., 2007; Ma et al., 2007; Cui et al., 2012; Xu et al., 2014; Liu et al., 2016; Zhai et al., 2016), primarily involving chromosomes 1A, 1B, 2D, 3A, 3B, 4A, 4B, 5A, 5D, 7A, and 7D. In our study, stable QTLs for Ssn and Fsn, which were not affected by N application at the different stages, were found in the same intervals on chromosomes 1B, 2B, 2D, and 5D. The stable QTLs *QSsn.sdau-2B-1* and *QFsn.sdau-2B-1* on chromosome 2B are particularly noteworthy because they are novel QTLs and are first reported herein. Additionally, the stable QTL *QGns.sdau-2B* in the same region was first detected in this study. Gns was significantly positively correlated with Fsn but negatively correlated with Ssn. These results indicate that the loci controlling these three traits are often not independent and that increasing Fsn, decreasing Ssn or both could improve Gns, which is consistent with practical breeding experience. The marker interval *Xwmc112*-*Xcfd53*-*Xwmc18* on chromosome 2D with pleiotropic effects was found to involve six QTLs, including QTLs for not only Sl but also Ssn and Fsn. Cui et al. (2012) and Zhai et al. (2016) also identified QTLs for Sl, Ssn and Fsn. Located in this region is the gene Rht8, which has been associated with QTLs for plant height, Sl, spikelet number, Tkw, spikelet compactness and grain yield (Ma et al., 2007; Cui et al., 2012; Xu et al., 2014; Zhai et al., 2016). Therefore, this region will be important for improving spike-related traits. In addition, Li et al. (2007) found only one QTL, *QFss.sdau-5D.e3*, for Fsn, with 70.25% PVE, on chromosome 5D in one environment. Similarly, we detected a QTL for Fsn on chromosome 5D in this research but in a different region, i.e., in the *Xbarc320*-*Xwmc215* interval. Some important QTLs for heading date and chlorophyll content are also associated with this region (Zhang et al., 2009a,b). These results suggest that this region is important for spike development.

In previous studies, most QTLs for Tkw have been detected on chromosomes 1B, 1D, 2A, 2B, 4B, 5A, 6A, 7B, and 7D (Huang et al., 2004; Li et al., 2007; Sun et al., 2009; Wang et al., 2009, 2011; Tsilo et al., 2010; Liu et al., 2016). Wang et al. (2011) detected a QTL in the marker interval *barc1055*-*barc37* on chromosome 6A in 237 F_2:3_ families derived from the cross of 3228 and Jing 4838. In the present study, we also found a stable QTL, *QTkw.sdau-6A*, between the markers *Xbarc1055* and *Xwmc553* on chromosome 6A, and importantly, *Xbarc1055* was found to be a common marker, indicating that they might be the same QTL. Moreover, this region contains an important gene controlling Tkw. Ding et al. (2011) and Liu et al. (2016) also identified QTLs for Tkw on chromosome 6A but at different positions than found here.

In addition, QTLs for nitrogen concentration and utilization efficiency traits have been reported on chromosomes 3A, 4B, 4D, 6A, 5A, and 7A (Xu et al., 2014). Cormier et al. (2014) also identified the important chromosomal regions determining nitrogen use efficiency components using a genome-wide association study, involving chromosomes 2D, 4D, 1A, 3A, 3B, 4B, 2A, 5B, and 7B for related traits, such as nitrogen use efficiency, nitrogen use to protein efficiency, nitrogen utilization efficiency, nitrogen utilization to protein efficiency, nitrogen uptake, nitrogen harvest index, straw dry matter at maturity, straw nitrogen content at maturity. In our study, we also found several QTLs on chromosomes 2A, 2D, 4B, 5A, 5B, 6A, and 7B when nitrogen fertilization was used, such as *QSl.sdau-2A*, QSsn.sdau-5A, *QTkw.sdau-5B, QSl.sdau-7B*, etc., which perhaps were induced by nitrogen treatment. Moreover, there were also some QTLs identified on other chromosomes, such as 1B, 2B, 4A, 5D, and 7D. Of these QTL, *QSsn.sdau-1B-1, QSsn.sdau-5D, QFsn.sdau-5D* were detected in at least 2 years and two nitrogen treatments. Several publications already mentioned the important region on chromosomes 2D as affecting plant height, Sl, Ssn per spike, and harvest index (Cormier et al., 2014; Xu et al., 2014), this region was identical to gene Rht8 (Korzun et al., 1998; Worland et al., 1998; Zhai et al., 2016), and perhaps also related to photoperiod gene. The QTL on chromosome 6A for Tkw in this study had the same marker Xbarc1055 with the QTLs *QNUtE*_*DM*_*-6A* and *QNUtE*_*GY*_*-6A* for nitrogen utilization efficiency for aboveground dry matter and grain yield (Xu et al., 2014). This region also affected NutE_Prot (nitrogen use to protein efficiency), grain number per ear, root dry weight and %N_S (straw nitrogen content at maturity) (An et al., 2006; Habash et al., 2007; Xu et al., 2014). Its interval was related to the genes *TaGW2* and glutamine synthetase *GS 1*. Therefore, these new QTLs identified under each different treatment perhaps were important for reasonable cultivation from molecular level because of relating to important genes for nitrogen use efficiency-related traits. In the future, to dissect the influence of cultivation treatments on QTL expression, the conditional QTL mapping should be used besides unconditional QTL mapping.

In summary, 70 QTLs involving five spike-related traits were detected in different environments, and eight critical chromosome regions were found. Sixteen stable QTLs that were little affected by N application treatments were identified. Of these, the flanking markers of important QTLs on chromosomes 2B, 2D, and 6A for Ssn, Fsn, Sl, and Tkw can be used in MAS breeding. The results of this study increase our understanding of the genetic basis of spike-related traits.

## Author contributions

ZD analyzed the data and wrote the manuscript; YC and JL investigated the phenotypic data; QH and WF mapped the traits; and JT conceived the research and improved the manuscript. All authors carried out the field experiments and have read and approved this manuscript.

### Conflict of interest statement

The authors declare that the research was conducted in the absence of any commercial or financial relationships that could be construed as a potential conflict of interest.

## References

[B1] An D. Su J. Liu Q. Zhu Y. Tong Y. Li J. (2006). Mapping QTLs for nitrogen uptake in relation to the early growth of wheat (*Triticum aestivum* L.). Plant Soil. 284, 73–84. 10.1007/s11104-006-0030-3

[B2] Börner A. Schumann E. Fürste A. Cöster H. Leithold B. Röder M.. (2002). Mapping of quantitative trait loci determining agronomic important characters in hexaploid wheat (*Triticum aestivum* L.). Theor. Appl. Genet. 105, 921–936. 10.1007/s00122-002-0994-1 12582918

[B3] Bremner J. M. (1960). Determination of nitrogen in soil by the Kjeldahl method. J. Agric. Sci. 55, 11–33. 10.1017/S0021859600021572

[B4] Chu C. G. Xu S. S. Friesen T. L. Faris J. D. (2008). Whole genome mapping in a wheat doubled haploid population using SSRs and TRAPs and the identification of QTL for agronomic traits. Mol. Breed. 22, 251–266. 10.1007/s11032-008-9171-9

[B5] Cormier F. Gouis J. L. Dubreuil P. Lafarge S. Praud S. (2014). A genome-wide identification of chromosomal regions determining nitrogen use efficiency components in wheat. Theor. Appl. Genet. 127, 2679–2693. 10.1007/s00122-014-2407-7 25326179

[B6] Cui F. Ding A. Li J. Zhao C. Wang L. Wang X. (2012). QTL detection of seven spike-related traits and their genetic correlations in wheat using two related RIL populations. Euphytica 186, 177–192. 10.1007/s10681-011-0550-7

[B7] Deng S. Wu X. Wu Y. Zhou R. Wang H. Jia J.. (2011). Characterization and precise mapping of a QTL increasing spike number with pleiotropic effects in wheat. Theor. Appl. Genet. 122, 281–289. 10.1007/s00122-010-1443-1 20872211

[B8] Deng X. F. Zhou Y. H. Yang R. W. Ding C. B. Zhang L. Zhang H. Q. (2005). Chromosomal location of genes for spike length in dwarfing polish wheat by monosomic analysis. J. Sichun Agric. Univ. 23, 12–14.

[B9] Ding A. M. Li J. Cui F. Zhao C. H. Ma H. Y. Wang H. G. (2011). QTL mapping for yield related traits using two associated RIL populations of wheat. Acta Agron. Sin. 37, 1511–1524. 10.1016/S1875-2780(11)60041-2

[B10] Doerge R. W. (2002). Multifactorial genetics: mapping and analysis of quantitative trait loci in experimental populations. Natl. Rev. 3, 43–52. 10.1038/nrg703 11823790

[B11] Gorjanović B. Kraljević-Balalić M. (2005). Inheritance of plant height and spike length in wheat. Genetika 37, 25–31. 10.2298/GENSR0501025G

[B12] Gorjanovic B. Kraljevic-Balalic M. (2007). Inheritance of plant height, spike length and number of spikelets per spike in Durum wheat. Zbornik Matice Srpske Za Prirodne Nauke 112, 27–33. 10.2298/ZMSPN0712027G

[B13] Gupta P. K. Langridge P. Mir R. R. (2010). Marker-assisted wheat breeding: present status and future possibilities. Mol. Breed. 26, 145–161. 10.1007/s11032-009-9359-7

[B14] Habash D. Z. Bernard S. Schondelmaier J. Weyen J. Quarrie S. A. (2007). The genetics of nitrogen use in hexaploid wheat: Nutilisation, development and yield. Theor. Appl. Genet. 114, 403–419. 10.1007/s00122-006-0429-5 17180378

[B15] Huang X. Q. Cloutier S. Lycar L. Radovanovic N. Humphreys D. G. Noll J. S.. (2006). Molecular detection of QTLs for agronomic and quality traits in a doubled haploid population derived from two Canadian wheats (*Triticum aestivum* L.). Theor. Appl. Genet. 113, 753–766. 10.1007/s00122-006-0346-7 16838135

[B16] Huang X. Q. Kempf H. Ganal M. W. Roder M. S. (2004). Advanced backcross QTL analysis in progenies derived from a cross between a German elite winter wheat variety and a synthetic wheat (*Triticum aestivum* L.). Theor. Appl. Genet. 109, 933–943. 10.1007/s00122-004-1708-7 15243706

[B17] Ijaz U. S. Kashif M. (2013). Genetic study of quantitative traits in spring wheat through generation means analysis. Am. Eurasian J. Agric. Environ. Sci. 13, 191–197. 10.5829/idosi.aejaes.2013.13.02.1101

[B18] Islamovic E. Obert D. E. Oliver R. E. Marshall J. M. Miclaus K. J. Hang A. (2013). A new genetic linkage map of barley (*Hordeum vulgare* L.) facilitates genetic dissection of height and spike length and angle. Field Crops Res. 154, 91–99. 10.1016/j.fcr.2013.06.001

[B19] Jantasuriyarat C. Vales M. I. Watson C. J. Riera-Lizarazu O. (2004). Identification and mapping of genetic loci affecting the free-threshing habit and spike compactness in wheat (*Triticum aestivum* L.). Theor. Appl. Genet. 108, 261–273. 10.1007/s00122-003-1432-8 13679977

[B20] Korzun V. Röder M. Ganal M. Worland A. Law C. (1998). Genetic analysis of the dwarfing gene (*Rht8*) in wheat. Part I. Molecular mapping of *Rht8* on the short arm of chromosome 2D of bread wheat (*Triticum aestivum* L.). Theor. Appl. Genet. 96, 1104–1109. 10.1007/s001220050845

[B21] Kumar N. Kulwal P. L. Balyan H. S. Gupta P. K. (2007). QTL mapping for yield and yield contributing traits in two mapping populations of bread wheat. Mol. Breed. 19, 163–177. 10.1007/s11032-006-9056-8

[B22] Li S. Jia J. Wei X. Zhang X. Li L. Chen H. (2007). A intervarietal genetic map and QTL analysis for yield traits in wheat. Mol. Breed. 20, 167–178. 10.1007/s11032-007-9080-3

[B23] Liu K. Deng Z. Y. Li Q. F. Zhang Y. Sun C. L. Tian J. C. (2016). Mapping QTLs for wheat panicle traits with high density SNP genetic map. Acta Agron. Sin. 42, 820–831. 10.3724/SP.J.1006.2016.00820

[B24] Lu X. Zhang J. Wang H. Yang X. Li X. Li L. (2011). Genetic analysis and QTL mapping of wheat spike traits in a derivative line 3558 from wheat x *Agronpyron cristatum* offspring. J. Plant Gene.t Resour. 12, 86–91.

[B25] Ma Z. Zhao D. Zhang C. Zhang Z. Xue S. Lin F.. (2007). Molecular genetic analysis of five spike-related traits in wheat using RIL and immortalized F2 populations. Mol. Genet. Genomics 277, 31–42. 10.1007/s00438-006-0166-0 17033810

[B26] Madiš M. Kneţeviš D. Paunoviš A. Duroviš D. Jeliš M. (2010). Inheritance of stem height and primary spike length in barley hybrids, in Proceedings of 45th Croatian and 5th International Symposium on Agriculture, Genetics, Plant Breeding and Seed Production, ed Maric S. Lončaric Z. Maric S. Lončaric Z. (Opatija), 56–460.

[B27] Marza F. Bai G. H. Carver B. F. Zhou W. C. (2006). Quantitative trait loci for yield and related traits in the wheat population Ning7840 x Clark. Theor. Appl. Genet. 112, 688–698. 10.1007/s00122-005-0172-3 16369760

[B28] Melich A. (1953). Determination of P, Ca, Mg, K, Na and NH 4. North Carolina Soil Testing Division. North Carolina State University (Raleigh, NC).

[B29] Miura H. Parker B. B. Snape J. W. (1992). The location of major genes and associated quantitative trait loci on chromosome arm 5BL of wheat. Theor. Appl. Genet. 85, 197–204. 10.1007/BF00222860 24197305

[B30] Nataša L. Sofija P. Miodrag D. Nikola H. Mirjana V. Zorana S. (2014). Diallel analysis for spike length in winter wheat. Turk. J. Agric. Nat. Sci. Special. 2, 1455–1459.

[B31] Sharma S. N. Sain R. S. Sharma R. K. (2003). Genetics of spike length in durum wheat. Euphytica 130, 155–161. 10.1023/A:1022814301871

[B32] Sun X. Y. Wu K. Zhao Y. Kong F. M. Han G. Z. Jiang H. M. (2009). QTL analysis of kernel shape and weight using recombinant inbred lines in wheat. Euphytica 165, 615–624. 10.1007/s10681-008-9794-2

[B33] Tsilo T. J. Hareland G. A. Simsek S. Chao S. Anderson J. A. (2010). Genome mapping of kernel characteristics in hard red spring wheat breeding lines. Theor. Appl. Genet. 121, 717–730. 10.1007/s00122-010-1343-4 20425103

[B34] Walkley A. Black I. A. (1934). An examination of the Degtjareff method for determining soil organic matter and a proposed modification of the chromic acid titration method. Soil Sci. 37, 29–38. 10.1097/00010694-193401000-00003

[B35] Wang J. (2009). Inclusive composite interval mapping of quantitative trait genes. Acta Agron. Sin. 35, 239–245. 10.3724/SP.J.1006.2009.00239

[B36] Wang J. Liu W. Wang H. Li L. Wu J. Yang X. (2011). QTL mapping of yield-related traits in the wheat germplasm 3228. Euphytica 177, 277–292. 10.1007/s10681-010-0267-z

[B37] Wang R. X. Hai L. Zhang X. Y. You G. X. Yan C. S. Xiao S. H. (2009). QTL mapping for grain filling rate and yield-related traits in RILs of the Chinese winter wheat population Heshangmai x Yu8679. Theor. Appl. Genet. 118, 313–325. 10.1007/s00122-008-0901-5 18853131

[B38] Worland A. Korzun V. Röder M. Ganal M. Law C. (1998). Genetic analysis of the dwarfing gene (*Rht8*) in wheat. Part II. The distribution and adaptive significance of allelic variants at the *Rht8* locus of wheat as revealed by microsatellite screening. Theor. Appl. Genet. 96, 1110–1120. 10.1007/s001220050846

[B39] Wu X. Cheng R. Xue S. Kong Z. Wan H. Li G. (2014). Precise mapping of a quantitative trait locus interval for spike length and grain weight in bread wheat (*Triticum aestivum* L.). Mol. Breed. 33, 129–138. 10.1007/s11032-013-9939-4

[B40] Xu Y. Wang R. Tong Y. Zhao H. Xie Q. Liu D.. (2014). Mapping QTLs for yield and nitrogen-related traits in wheat: influence of nitrogen and phosphorus fertilization on QTL expression. Theor. Appl. Genet. 127, 59–72. 10.1007/s00122-013-2201-y 24072207

[B41] Yang R. Liu L. Li H. Zhong H. Yang X. Wang Z. (2012). QTL analysis of spike traits in an recombinant inbred lines (RILs) population derived from the cross of *Triticum polonicum* × T. *aestivum* line Zhong 13. J. Agric. Biotechnol. 20, 506–513. 10.3969/j.issn.1674-7968.2012.05.006

[B42] Yu M. Mao S. L. Chen G. Y. Pu Z. E. Wei Y. M. Zheng Y. L. (2014). QTLs for uppermost internode and spike length in two wheat RIL populations and their affect upon plant height at an individual QTL level. Euphytica 200, 95–108. 10.1007/s10681-014-1156-7

[B43] Yuan A. Cai C. Lou X. Gao M. (1997). Analysis on the genetic model of spike length of wheat main axic. J. Luoyang Agric. Coll. 17, 19–22.

[B44] Yuen S. H. Pollard A. G. (1953). Determination of nitrogen in soil and plant materials: use of boric acid in the micro-Kjeldahl method. J. Sci. Food Agric. 4, 490–496. 10.1002/jsfa.2740041006

[B45] Zadoks J. C. Chang T. T. Konzak C. F. (1974). A decimal code for the growth stages of cereals. Weed Res. 14, 415–421. 10.1111/j.1365-3180.1974.tb01084.x

[B46] Zandstra H. G. (1968). Automated determination of phosphorus in sodium bicarbonate extracts. Can. J. Soil Sci. 48, 219–220. 10.4141/cjss68-029

[B47] Zhai H. Feng Z. Li J. Liu X. Xiao S. Ni Z.. (2016). QTL analysis of spike morphological traits and plant height in winter wheat (*Triticum aestivum* L.) using a high-density SNP and SSR-based linkage map. Front. Plant Sci. 7:1617. 10.3389/fpls.2016.01617 27872629 PMC5097907

[B48] Zhang K. P. Zhao L. Tian J. C. Chen G. F. Jiang X. L. Liu B. (2008). A genetic map conducted using a doubled haploid population derived from two elite Chinese common wheat (*Triticum aestivum* L.) varieties. J. Integr. Plant Biol. 50, 941–950. 10.1111/j.1744-7909.2008.00698.x 18713343

[B49] Zhang K. Tian J. Zhao L. Liu B. Chen G. (2009a). Detection of quantitative trait loci for heading date based on the doubled haploid progeny of two elite Chinese wheat cultivars. Genetica 135, 257–265. 10.1007/s10709-008-9274-6 18500653

[B50] Zhang K. Zhang Y. Chen G. Tian J. (2009b). Genetic analysis of grain yield and leaf chlorophyll content in common wheat. Cereal Res. Commun. 37, 499–511. 10.1556/CRC.37.2009.4.3

[B51] Zheng Y. Yan J. Yang J. (1992). Location of genes for spike length in common wheat. J. Sichun Agric. Univ. 10, 570–573.

