# Supplementary material for: Discovery of Consistent QTLs of Wheat Spike-Related Traits under Nitrogen Treatment at Different Development Stages
Source: Front Plant Sci. 2017 Dec 15;8:2120. doi: 10.3389/fpls.2017.02120 (PMC5737097; doi:10.3389/fpls.2017.02120)

**Figure S1.** Distribution of Five Spike-Related Traits under Four Different Treatments in Two Locations in Two Years.

Explanations: The unit on Y axes in all the figures is ‘%’.

The unit on X axes in the figures for Gns is ‘the number of grains per spike’.

The unit on X axes in the figures for Ssn and Fsn is ‘the number per spike’.

The unit on X axes in the figures for Tkw is ‘g’.

The unit on X axes in the figures for Sl is ‘cm’.


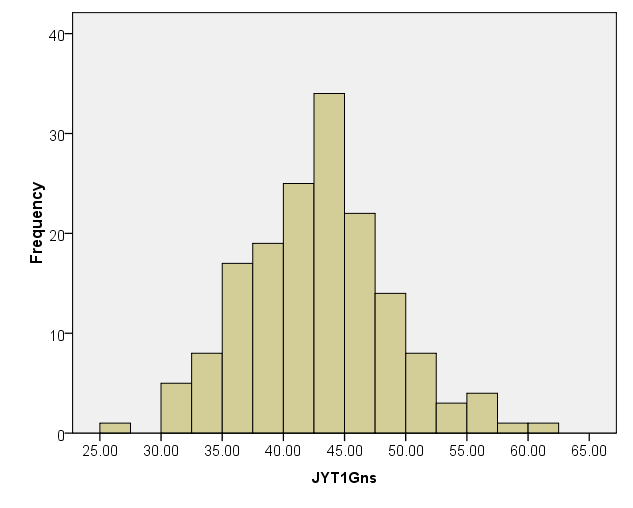

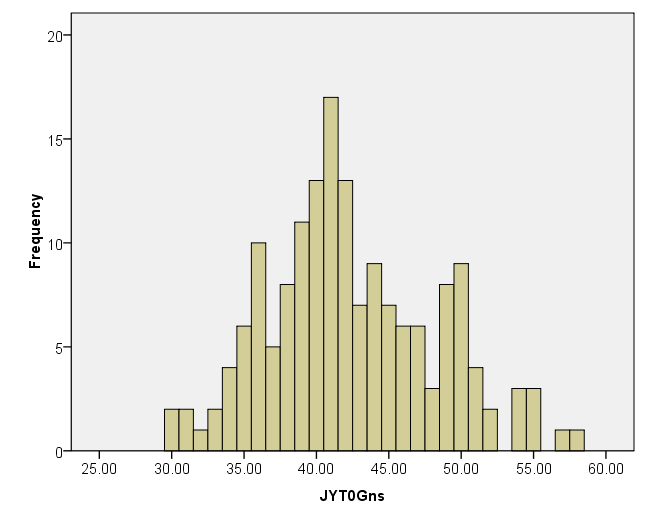


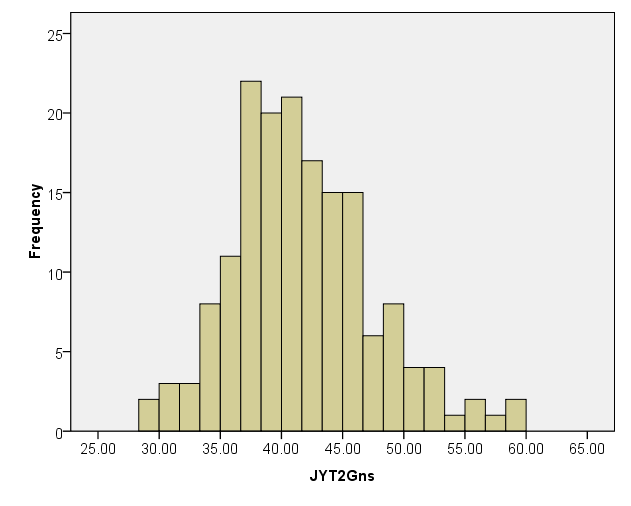


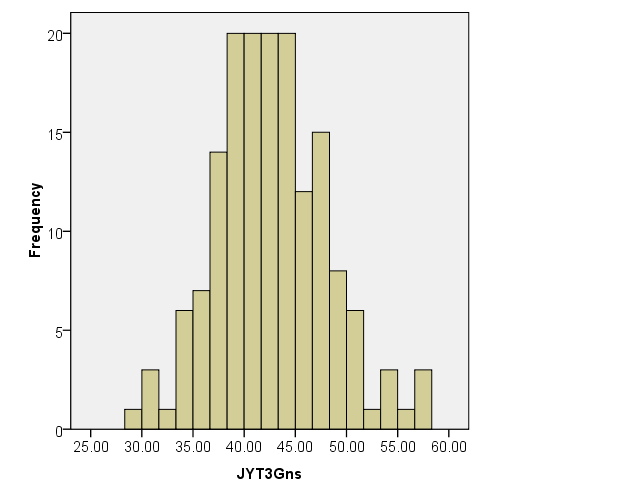


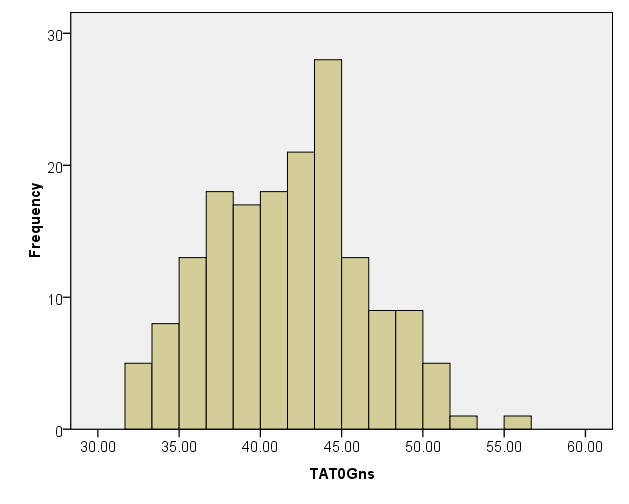

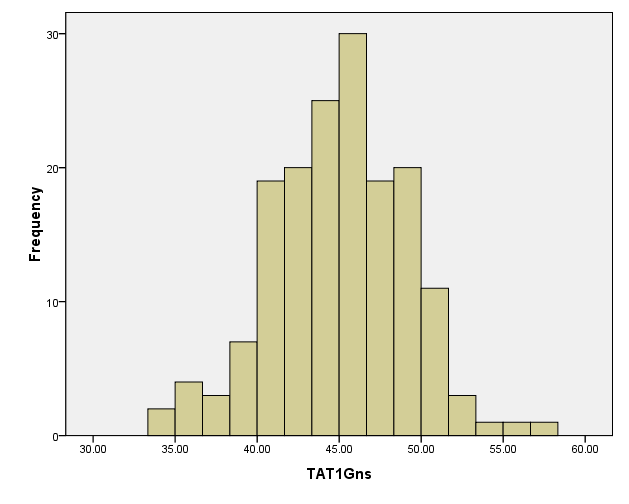


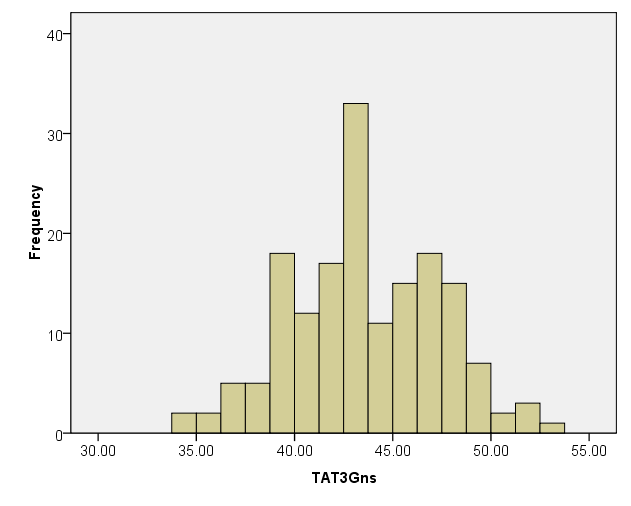

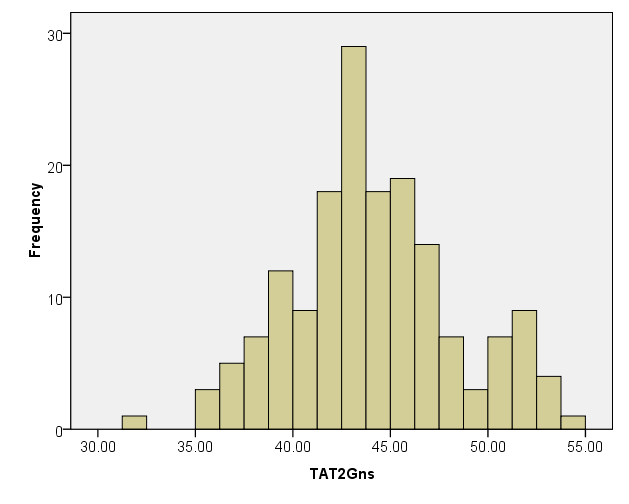


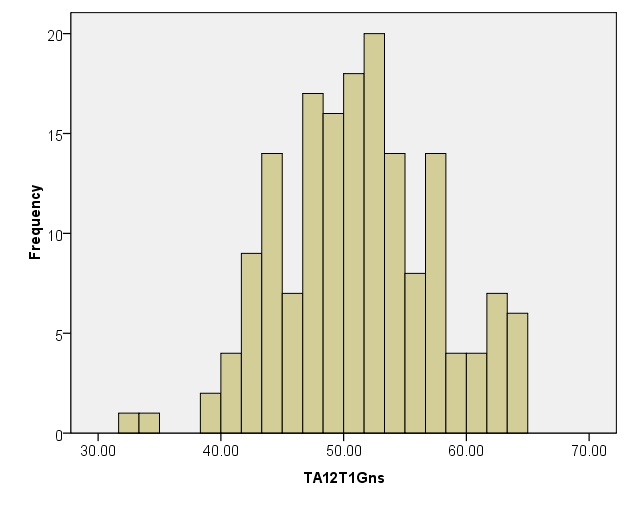

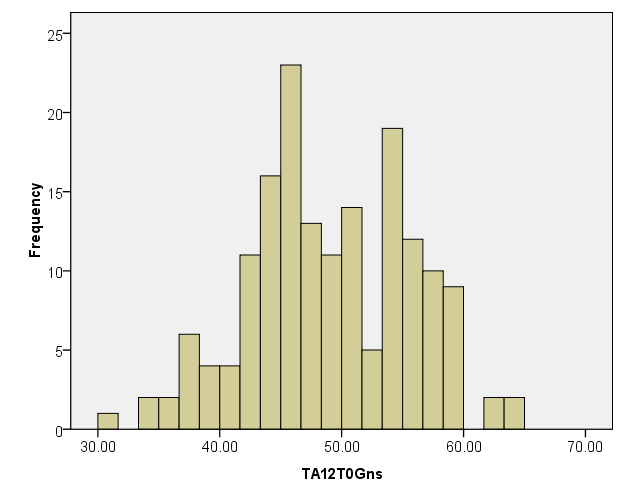


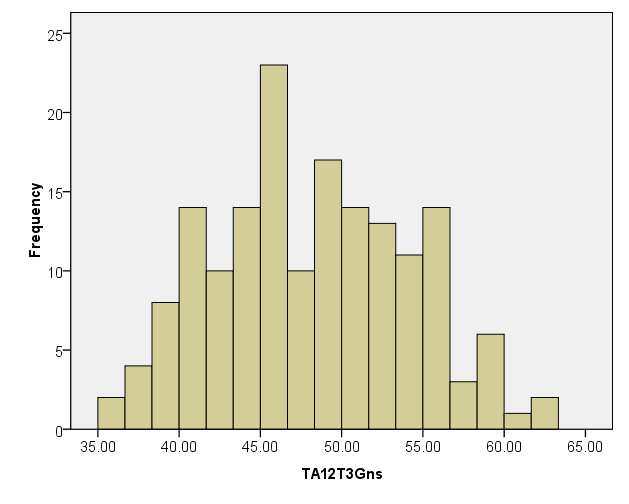

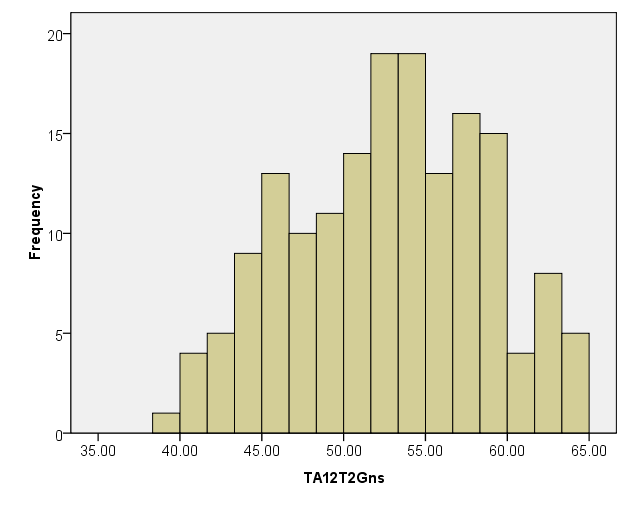

Supplement: Supplementary file 1 [file DataSheet1.DOCX]
